# A QSP model of prostate cancer immunotherapy to identify effective combination therapies

**DOI:** 10.1038/s41598-020-65590-0

**Published:** 2020-06-03

**Authors:** Roberta Coletti, Lorena Leonardelli, Silvia Parolo, Luca Marchetti

**Affiliations:** 1 University of Trento, Department of mathematics, Trento, 38123 Italy; 2 Fondazione The Microsoft Research - University of Trento Centre for Computational and Systems Biology (COSBI), Rovereto, 38068 Italy

**Keywords:** Cancer immunotherapy, Prostate cancer, Computational models, Differential equations, Prostate cancer

## Abstract

Immunotherapy, by enhancing the endogenous anti-tumor immune responses, is showing promising results for the treatment of numerous cancers refractory to conventional therapies. However, its effectiveness for advanced castration-resistant prostate cancer remains unsatisfactory and new therapeutic strategies need to be developed. To this end, systems pharmacology modeling provides a quantitative framework to test *in silico* the efficacy of new treatments and combination therapies. In this paper we present a new Quantitative Systems Pharmacology (QSP) model of prostate cancer immunotherapy, calibrated using data from pre-clinical experiments in prostate cancer mouse models. We developed the model by using Ordinary Differential Equations (ODEs) describing the tumor, key components of the immune system, and seven treatments. Numerous combination therapies were evaluated considering both the degree of tumor inhibition and the predicted synergistic effects, integrated into a decision tree. Our simulations predicted cancer vaccine combined with immune checkpoint blockade as the most effective dual-drug combination immunotherapy for subjects treated with androgen-deprivation therapy that developed resistance. Overall, the model presented here serves as a computational framework to support drug development, by generating hypotheses that can be tested experimentally in pre-clinical models.

## Introduction

Mathematical modeling has been successfully applied in the context of computational systems biology to develop comprehensive mathematical descriptions of several pathologies^1–9^. Mathematical models are calibrated through experimental data to simulate *in silico* biological systems and test hypotheses, for example regarding the regulative mechanisms of complex diseases^10–16^. Quantitative system pharmacology (QSP) is a popular modeling approach that supports the pharmaceutical industry in validating or identifying drug targets, designing new therapies and evaluating side effects^17–21^. QSP models allow the inclusion of several data from different sources, integrated in compartmental and hierarchical mathematical models. This approach tests different therapeutic protocols, studying drug responses and effects as well as understanding in a more accurate way the mechanisms behind a modeled phenomenon^22^. In this paper, the QSP modeling approach is applied to cancer immunotherapy^23^, a very promising medical area of growing interest.

In cancer immunotherapy, therapeutic agents are used to enhance the host anti-tumour immune response by perturbing the tumor microenvironment^24^. The idea of modulating the immune response as cancer therapy is a direct consequence of the strong, well recognized interplay between tumors and the immune system. Indeed, the genetic alterations in cancer cells promote the activation of the immune system that starts a series of events, known as cancer-immunity cycle, to control cancer growth^25^. However, the tumor can develop several mechanisms to escape the immune control, such as the inactivation of Cytotoxic T Lymphocytes (CTLs) and the recruitment of immune suppressor cells^25,26^, mainly Regulatory T cells (Tregs)^27,28^ and Myeloid-Derived Suppressor Cells (MDSCs)^29–31^. Therefore, the availability of immuno-therapeutic agents reinforcing the cancer-immunity cycle is crucial for a successful treatment outcome. Over the past few years, different types of immunotherapies have been developed, spanning from Immune-Checkpoint Blockade (ICB) to strategies that boost the T cell activity^32^.

In this work, we focus on Prostate Cancer (PCa), one of the leading causes of cancer-associated death in the male population^33–35^. Patients diagnosed with localized PCa are usually monitored for their blood levels of prostate-specific antigen and, when appropriate, are treated with radiation therapy or prostatectomy^36^. However, 20–40% of patients develops PCa recurrence and requires further treatments^37–39^. Since PCa cell proliferation is dependent on androgen signaling, androgen deprivation therapy, either by chemical or surgical castration, is the first-line treatment for advanced PCa^40^. Although this therapy is initially highly effective in most of the patients, in some cases the tumor evolves into an androgen independent form that currently lacks efficacious therapeutic options^41^. The transition from the androgen dependent to the androgen independent state may occur through several mechanisms that are not yet completely understood^42^.

In this context, immunotherapy represents a highly promising new treatment approach. Over the last few years, numerous pre-clinical and clinical studies have been performed to develop and test different PCa immunotherapies^43^. A main achievement has been the US Food and Drug Administration (FDA) approval of sipuleucel-T, so far the only approved immunotherapy for PCa treatment^40^. Compared with other types of cancer, PCa is relatively insensitive to the most popular immunotherapies and additional studies are needed to understand the mechanisms underlying this lack of immune responsiveness^44^. Thus, evaluating combination therapies is another important step to improve therapeutic benefits^45^.

We herein propose a QSP model of prostate cancer that extends a previous one published by Peng *et al*.^46^ based on data from a murine Pten prostate cancer model^47,48^.

Although Peng and coworkers already described the action of CTLs, dendritic cells, Tregs, androgens and interleukin-2 (IL-2) in the tumor microenvironment, we extended the characterization of the immune system by including MDSCs and Natural Killer (NK) cells as potential targets for new prostate cancer therapies. The main goal of this contribution is, indeed, to provide a mathematical model able to test the efficacy of several immunotherapies and their combinations. We incorporate a wide range of experimental data from literature^31,46,49–51^ to implement seven treatments. The efficacy of therapies is assessed considering the model-predicted tumor inhibitory effect and the synergy of combination therapies. Synergy between treatments plays a crucial role to reduce the dosage of each drug maintaining a satisfactory overall treatment efficacy, improving patients’ quality of life^52^.

The paper is organized as follows. In the result section we provide a description of the model diagram and a detailed explanation of the Ordinary Differential Equations (ODEs) constituting the mathematical model. We provide the model simulations to verify the reproducibility of the experimental data and to compare the predicted outcomes with known biological mechanisms. We identify the most effective treatment combinations for prostate cancer through the model-predicted tumor size and the treatment synergy. Moreover, we emphasize the androgen deprivation therapy as leading treatment in a decision tree to choose the best protocol to treat androgen independent prostate cancer. Finally, a discussion of the results and a description of the study limitations is provided.

## Results

### The mathematical model

**Figure 1:**
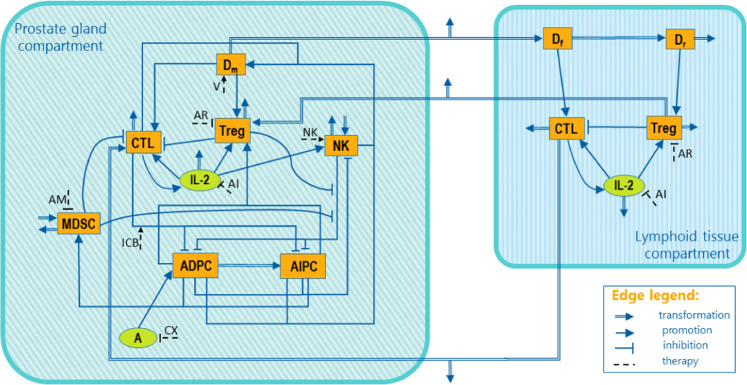
Model diagram. The model is composed of two compartments: prostate gland and lymphoid tissue. Cells are represented in orange squared boxes, while molecules in green rounded ones. In the prostate gland compartment the cancer is in its two forms: Androgen Dependent Prostate Cancer (ADPC) and Androgen Independent Prostate Cancer (AIPC). Other players involved: mature Dendritic cell (Dm), regulatory T cell (Treg), Myeloid-Derived Suppressor Cell (MDSC), Cytotoxic T Lymphocyte (CTL), Natural Killer cell (NK), functional Dendritic cells (Df), regulatory Dendritic cells (Dr), Androgen (A) and Interleukin-2 (IL-2). Double arrows represent transformations, single lines represent promotions and inhibitions, while dashed lines depict the seven implemented treatments. A complete description of the treatments and the corresponding abbreviations are reported in Table 1.

Starting from the model introduced by Peng *et al*.^46^, we built a mathematical model based on ODEs describing the prostate cancer and its interaction with the immune system. A graphical representation of the model variables and their regulative effects is shown in Fig. 1. The model has two compartments: the prostate gland and the lymphoid tissue. The prostate gland compartment includes Androgen Dependent (ADPC) and Independent (AIPC) Prostate Cancer cells. While the ADPC grows in the presence of androgens, under androgen deprivation therapy ADPC cells undergo apopotosis. However, the low androgen level leads to the AIPC proliferation. ADPC and AIPC cell expansion is counteracted by the activation of the host immune system. NK cells and CTLs have been included as the major effectors of the innate and adaptive immune response, respectively. NK and CTL killing activity is counteracted by Tregs and MDSCs, immune cells promoting immune tolerance. In addition, IL-2 has been included into the model as a key signaling molecule promoting the proliferation of CTLs, NK and Treg cells. The lymphoid tissue compartment includes variables representing biological processes of the prostate draining lymph nodes and the spleen. Within this compartment, functional (Df) and regulatory (Dr) dendritic cells activate CTLs and Treg cells, respectively. The two compartments communicate by exchanging dendritic cells, CTLs and Tregs. In addition, the model incorporates 7 different treatments, shown with dashed lines in Fig. 1 and summarized in Table 1.

**Table 1:** Summary of the treatments implemented in the mathematical model.

Therapy	Abbreviation	Description	Reference
castration	CX	androgen deprivation within the prostate gland	^46^
vaccine	V	administration of mature dendritic cells into the prostate gland	^46^
anti-IL2	AI	administration of monoclonal antibodies neutralizing IL-2 within the prostate gland and the lymphoid tissue	^46^
anti-Treg	AR	Treg depletion within the prostate gland and the lymphoid tissue through anti-CD25 antibody injection	^46^
NK cells	NK	administration of NK cells within the prostate gland implemented by increasing the initial condition of NK	^108^
Immune checkpoint blockade	ICB	an intermittent therapy with a cocktail of anti-CTLA4 and anti-PD1 antibodies administered three times per week, for 4 weeks, starting 21 days after androgen deprivation therapy	^51^
anti-MDSC	AM	an intermittent therapy with anti-MDSC drugs (Cabozatinib) daily administered for 4 weeks, starting 21 days after androgen deprivation therapy	^51^

In total, the model is composed of 19 ODEs, which describe the dynamics of 14 variables related to the tumor and the immune system, and 5 treatment-related variables. It is worth noting that the androgen deprivation therapy (CX) and the infusion of NK do not have an equation describing their behaviour. The androgen deprivation is included in the model as a term of the Eq. (9), while the infusion of NK is implemented by changing the initial condition of NK cells. Compared with the starting model^46^, our extended version includes two additional variables describing the tumor microenvironment (NK cells and MDSC) and two variables related to the treatment: the immune checkpoint blockade (ICB) and the MDSC-targeted therapy (AM). Moreover, the original equations have been extended to take into account the action of the new players. Each variable is expressed as a ratio between its current value and its initial value.

The model equations are described in the following subsections, while the model parameters are reported in Supplementary Table S1. Before calibrating the model, we also evaluated its structural identifiability by means of the Matlab toolbox GenSSI^53^. Despite some limitations of the employed algorithm, which imposed some restrictions on the amount of experimental data that was specified as being used during the model calibration phase, the analysis guarantees a local identifiability. The Matlab sources needed to run the analysis are provided in the Supplementary File 2, we refer to the corresponding section of Materials and Methods for further details.

#### Prostate gland compartment

The prostate gland compartment contains prostate cancer cells (ADPC and AIPC), immune system cells (Dm, CTL, NK, Treg and MDSC), androgens and IL-2.

1 $$\begin{array}{rcl}\frac{d{X}_{1}}{dt} & = & \mathop{\underbrace{{r}_{p1}\,A\,{X}_{1}}}\limits_{{\rm{proliferation}}}\mathop{\underbrace{-{\mu }_{1}\,\mathrm{(1}-A)\,{X}_{1}}}\limits_{{\rm{death}}}\mathop{\underbrace{-{r}_{M}\,\mathrm{(1}-A)\,{X}_{1}}}\limits_{{\rm{mutation}}}\\  &  & \mathop{\underbrace{-{k}_{CX}\,\mathrm{(1}+{\delta }_{ICB}\,{I}_{CB})\,{C}_{2}\,{X}_{1}}}\limits_{{\rm{tumor}}\,{\rm{killing}}\,{\rm{by}}\,{\rm{CTL}}}\mathop{\underbrace{-{k}_{NX}({e}^{-{k}_{RN}{R}_{2}}+{e}^{-{k}_{MN}M})\,N\,{X}_{1}}}\limits_{{\rm{tumor}}\,{\rm{killing}}\,{\rm{by}}\,{\rm{NK}}}\end{array}$$

The Eq. (1) refers to the ADPC cells. Tumor cell proliferation is positively regulated by the androgen presence, while tumor death and mutation are negatively affected by it. We assume that the evolution to AIPC occurs after androgen deprivation therapy, as a selective pressure is applied (“mutation” term). A key mechanism exploited by the tumor to evade the immuno-surveillance is the immune checkpoint activation, which reduces the CTL tumor-killing capacity^25^. ICB treatments are used to restore the T cell effector functions^54^. In our model, this biological process is described by the linear function $1+{\delta }_{ICB}\,{I}_{CB}$ in the “tumor killing by CTL” term, in which the ${I}_{CB}$ represents the amount of ICB drug (Eq. (19)). This function increases the value of the CTL killing capacity (${k}_{CX}$) when the treatment is administered. The “tumor killing by NK” term depends on a multiplicative function of Treg (${R}_{2}$) and MDSC cells (*M*)^55^. Following the approach of De Pillis *et al*.^56^, this inhibition has been modeled by a sum of two negative exponential terms, which becomes close to zero when the amount of Treg or MDSC increases.

2 $$\begin{array}{rcl}\frac{d{X}_{2}}{dt} & = & \mathop{\underbrace{{r}_{p2}\,{X}_{2}}}\limits_{{\rm{proliferation}}}\mathop{\underbrace{-{\mu }_{2}\,{X}_{2}}}\limits_{{\rm{death}}}\mathop{\underbrace{+{r}_{M}\mathrm{(1}-A)\,{X}_{1}}}\limits_{mutation}\\  &  & \mathop{\underbrace{-{k}_{CX}\mathrm{(1}+{\delta }_{ICB}{I}_{CB})\,{C}_{2}\,{X}_{2}}}\limits_{{\rm{tumor}}\,{\rm{killing}}\,{\rm{by}}\,{\rm{CTL}}}\mathop{\underbrace{-{k}_{NX}({e}^{-{k}_{RN}{R}_{2}}+{e}^{-{k}_{MN}M})\,N\,{X}_{2}}}\limits_{{\rm{tumor}}\,{\rm{killing}}\,{\rm{by}}\,{\rm{NK}}}\mathrm{}.\end{array}$$

The Eq. (2) describes the evolution in time of the AIPC cells. The terms of this equation are similar to the ones described above, except for proliferation and death terms, no longer androgen (*A*) dependent.

3 $$\begin{array}{rcc}\frac{d{D}_{m}}{dt} & = & \mathop{\underbrace{{a}_{VD}\,V}}\limits_{{\rm{vaccine}}}\mathop{\underbrace{+{a}_{XD}({\mu }_{1}\mathrm{(1}-A)\,{X}_{1}+{\mu }_{2}\,{X}_{2}+{k}_{CX}\mathrm{(1}+{\delta }_{ICB}{I}_{CB})\,{C}_{2}({X}_{1}+{X}_{2})+{k}_{NX}({e}^{-{k}_{RN}R}+{e}^{-{k}_{MN}M})N({X}_{1}+{X}_{2}))}}\limits_{{\rm{recruitment}}\,{\rm{by}}\,{\rm{tumor}}}\\  &  & \mathop{\underbrace{-{m}_{D}{D}_{m}}}\limits_{{\rm{migration}}}\mathrm{}.\end{array}$$

The mature dendritic cells are described in the Eq. (3). The amount of dendritic cells increases when the vaccine (V) is administered, as described by the vaccine term. The variable $V$ is 1 when vaccine is administered, $0$ otherwise. The dendritic cell recruitment and activation occur over tumor apoptosis^57,58^ and, once dendritic cells are activated (${D}_{m}$) within the inflammation site, they move to the lymphoid tissue compartment (${D}_{f}$ and ${D}_{r}$) as described by the migration term.

4 $$\begin{array}{rcl}\frac{d{C}_{2}}{dt} & = & \mathop{\underbrace{{a}_{DC}\,{D}_{m}}}\limits_{\mathop{{\rm{activation}}}\limits_{{\rm{by}}\,{{\rm{D}}}_{{\rm{m}}}}}\mathop{\underbrace{+{a}_{IC}\frac{{C}_{2}\,{I}_{2}}{{s}_{I}+{I}_{2}}}}\limits_{\mathop{{\rm{stimulation}}}\limits_{{\rm{by}}\,{\rm{IL}} \mbox{-} 2}}\mathop{\underbrace{+{p}_{C}\,{m}_{C}\,{C}_{1}}}\limits_{{\rm{migration}}}\\  &  & \mathop{\underbrace{-{k}_{RC}\,{R}_{2}\,{C}_{2}-{k}_{MC}\,M\,{C}_{2}}}\limits_{{\rm{inhibition}}\,{\rm{by}}\,{\rm{Treg}}\,{\rm{and}}\,{\rm{MDSC}}}\mathop{\underbrace{-{\mu }_{C}\,{C}_{2}}}\limits_{{\rm{death}}}\mathrm{}.\end{array}$$

The Eq. (4) describes the dynamics of the CTLs, which are activated by dendritic cells (${D}_{m}$) and IL-2 (${I}_{2}$)^59^. A fraction of CTLs migrates out from lymphoid tissue and reaches the prostate gland with a probability ${p}_{C}$ here set to 0.5^46^, as described by the “migration” term. The equation takes into account the effect of the immune-suppressive tumor microenvironment, here represented by the “inhibition by Treg and MDSC” term, and the CTL physiological cell death, as a negative regulation of CTL dynamics.

5 $$\frac{dN}{dt}=\mathop{\underbrace{{\rho }_{N}}}\limits_{{\rm{source}}}\mathop{\underbrace{-{\mu }_{N}N}}\limits_{{\rm{death}}}\mathop{\underbrace{-{k}_{XN}\,N({X}_{1}+{X}_{2})}}\limits_{{\rm{inhibition}}}\mathop{\underbrace{+{a}_{IN}\frac{N\,{I}_{2}}{{s}_{N}+{I}_{2}}}}\limits_{{\rm{stimulation}}\,{\rm{by}}\,{\rm{IL}} \mbox{-} 2}.$$

The Eq. (5) describes the NK cells dynamics. The source ${\rho }_{N}$ has been estimated by imposing a steady state when the tumor is not present (${X}_{1}+{X}_{2}=0$), assuming NK cells are not proliferating in absence of tumor-related inflammation. If we impose NK cells as constant, *i*.*e*. $\frac{dN}{dt}=0$, $N(0)=1$ and ${X}_{1}+{X}_{2}=0$, we obtain:

$${\rho }_{N}={\mu }_{N}-\frac{{a}_{IN}{I}_{2}}{{s}_{N}+{I}_{2}}.$$

Given the values of ${a}_{IN}$ and ${s}_{N}$ (Table S1), the ${\mu }_{N}$ parameter results as ${\mu }_{N}\gg \frac{{a}_{IN}{I}_{2}}{{s}_{N}+{I}_{2}}$, which leads to ${\rho }_{N}\approx {\mu }_{N}$.

For evaluating the death rate ${\mu }_{N}$, part of the death term, we followed the method used in de Pillis *et al*.^56^, where the authors considered the turnover rate of NK cells being inversely proportional to the term $\sqrt[4]{m}$, where $m$ is the body mass of the organism:

$${\mu }_{N}=\frac{K}{\sqrt[4]{m}}.$$

By considering the value of $k$ provided in the de Pillis *et al*.^56^ and by computing the mouse average mass^60,61^, we defined:

$${\mu }_{N}=\frac{0.0371}{\sqrt[4]{{m}_{mice}}}=0.09\,da{y}^{-1}.$$

The other terms of the equation describe regulatory mechanisms exerted by tumor and NK cells. Specifically, the “inhibition” term indicates NK inactivation after interaction with ADPC and AIPC, since PCa has the ability to impair NK cell function, establishing a strong immunosuppressive environment^62,63^. Moreover, PCa development has been associated with NK cell reduction^64^. The “stimulation by IL-2” term, instead, is the promotion exerted by IL-2 on NK cell proliferation^62,65^ within the prostate gland compartment.

6 $$\frac{d{R}_{2}}{dt}=\mathop{\underbrace{{a}_{DR}\,{D}_{m}}}\limits_{\begin{array}{c}{\rm{production}}\\ {\rm{by}}\,{{D}}_{{\rm{m}}}\end{array}}\mathop{\underbrace{+{a}_{IR}\,{I}_{2}}}\limits_{\begin{array}{c}{\rm{stimulation}}\\ {\rm{by}}\,{\rm{IL}}-2\end{array}}\mathop{\underbrace{+{p}_{R}\,{m}_{R}\,{R}_{1}}}\limits_{{\rm{migration}}}\mathop{\underbrace{+{a}_{XR}\,({X}_{1}+{X}_{2})}}\limits_{{\rm{activation}}\,{\rm{by}}\,{\rm{tumor}}}\mathop{\underbrace{-{\mu }_{R}\,{R}_{2}}}\limits_{{\rm{death}}}\mathop{\underbrace{-{k}_{antiRR}\,ant{i}_{R}\,{R}_{2}}}\limits_{{\rm{anti}}-{\rm{Treg}}\,{\rm{drug}}}\mathrm{}.$$

The Eq. (6) represents the Treg cells. Dendritic cell-mediated Treg production and IL-2 stimulation^27^ contribute to the amount of Treg in the prostate gland. The migration term instead accounts for the Treg cells migrating from the lymphoid tissue compartment. Following the same modeling approach used for CTL, these cells reach the prostate gland with a fixed probability of ${p}_{R}=0.5$^46^. “Activation by tumor” term describes the tumor ability to activate Treg cells as one of the tumor survival mechanisms^57^. Treg cells have a turnover rate of ${\mu }_{R}$, as shown in the death term of the equation. Treg cells are inactivated by the anti-Treg drug ($ant{i}_{R}$), which is set to 1 when administered, 0 otherwise.

7 $$\frac{dM}{dt}=\mathop{\underbrace{{\rho }_{M}}}\limits_{{\rm{source}}}\mathop{\underbrace{-{\mu }_{M}\,M}}\limits_{{\rm{death}}}\mathop{\underbrace{+{a}_{XM}\frac{({X}_{1}+{X}_{2})}{{s}_{M}+({X}_{1}+{X}_{2})}}}\limits_{{\rm{activation}}\,{\rm{by}}\,{\rm{tumor}}}\mathop{\underbrace{-{k}_{antiMM}\,ant{i}_{M}\,M}}\limits_{{\rm{anti}} \mbox{-} {\rm{MDSC}}\,{\rm{drug}}}\mathrm{}.$$

The Eq. (7) describes the MDSC dynamics in terms of MDSC source, death and tumor-dependent activation. The form of this equation is taken from Shariatpanahi *et al*.^66^. We adapted their parameter estimates to our non-dimensionalized model (Supplementary Table S1). The source of MDSCs is constant, while their turn-over is MDSC amount-dependent. Prostate cancer preserves its growth by recruiting and activating MDSC within the prostate microenvironment as described by the “activation by tumor” term. MDSC are inactivated by the anti-MDSC drug ($ant{i}_{M}$), which is set to 1 when administered, 0 otherwise.

8 $$\frac{d{I}_{2}}{dt}=\mathop{\underbrace{{a}_{CI}\,{C}_{2}}}\limits_{\mathop{{\rm{production}}}\limits_{{\rm{by}}\,{\rm{CTL}}}}\mathop{\underbrace{-{\mu }_{I}\,{I}_{2}}}\limits_{{\rm{death}}}\mathop{\underbrace{-{k}_{antiIl}\,ant{i}_{I}\,{I}_{2}}}\limits_{{\rm{anti}} \mbox{-} {\rm{IL}} \mbox{-} 2\,{\rm{drug}}}\mathrm{}.$$

The Eq. (8) describes the IL-2 dynamics. IL-2 is produced by several immune cells, mainly T cells^67^. As already modeled in other studies^46,56,68–70^, our model considers CTLs (${C}_{2}$) as the main responsible for IL-2 production, described by the “production by CTL” term.

In the death and anti-IL-2 drug terms, IL-2 physiological degradation rate ${\mu }_{I}$ is enhanced by the anti-IL-2 drug variable ($ant{i}_{I}$), which is $ant{i}_{I}\mathrm{(0)}=1$ in case of anti-IL-2 treatment.

9 $$\frac{dA}{dt}=\mathop{\underbrace{{\lambda }_{A}\mathrm{(1}-A)}}\limits_{\mathop{{\rm{proliferation}}}\limits_{{\rm{and}}\,{\rm{death}}}}\mathop{\underbrace{-{\lambda }_{A}\,{1}_{CX}}}\limits_{\mathop{{\rm{androgen}}}\limits_{{\rm{deprivation}}\,{\rm{effect}}}}\mathrm{}.$$

The Eq. (9) describes two possible androgen (*A*) dynamics, depending on the value of the Boolean function 1_*CX*_, which is set to 1 in case of androgen deprivation therapy, 0 otherwise.

When ${1}_{CX}=0$, the equation becomes:

$$\frac{dA}{dt}={\lambda }_{A}(1-A),$$

which is equal to zero by imposing the initial condition $A(0)=1$, *i*.*e*. constant androgen level over time.

Conversely, when ${1}_{CX}=1$, the androgen level exhibits an exponential decay with rate ${\lambda }_{A}$, calculated following the standard approach as:

$${\lambda }_{A}=\frac{ln\mathrm{(2)}}{{t}_{\mathrm{1/2}}},$$

where ${t}_{1/2}=7$ days is the androgen half-life^46^.

#### Lymphoid tissue compartment

The lymphoid tissue compartment contains the immune system cells (${D}_{f}$, ${D}_{r}$, CTLs and Tregs) and the IL-2.

10 $$\frac{d{D}_{f}}{dt}=\mathop{\underbrace{{p}_{D}\,{m}_{D}\,{D}_{m}}}\limits_{{\rm{migration}}}\mathop{\underbrace{-{a}_{DfDr}\,{D}_{f}}}\limits_{{\rm{transformation}}},$$

11 $$\frac{d{D}_{r}}{dt}=\mathop{\underbrace{{a}_{DfDr}\,{D}_{f}}}\limits_{{\rm{transformation}}}\mathop{\underbrace{-{\mu }_{D}\,{D}_{r}}}\limits_{{\rm{death}}}$$

Equations (10) and (11) represent the dynamics of the functional (${D}_{f}$) and regulatory (${D}_{r}$) dendritic cells, respectively. A fraction of ${D}_{m}$ cells migrates from the prostate gland to the lymphoid tissue, differentiating into functional dendritic cells with a probability ${p}_{D}$ here set to 0.5^46^ (“migration” term). A fraction of ${D}_{f}$ differentiates into ${D}_{r}$ within the lymphoid tissue compartment, as described by the “transformation” term, which reduces ${D}_{f}$ in Eq. (10) and increases ${D}_{r}$ in Eq. (11). ${D}_{r}$ cells are ultimately reduced by the death term in Eq. (11).

12 $$\frac{d{C}_{1}}{dt}=\mathop{\underbrace{{a}_{Df}\,{D}_{f}}}\limits_{\mathop{{\rm{activation}}}\limits_{{\rm{by}}\,{{\rm{D}}}_{{\rm{f}}}}}\mathop{\underbrace{+{a}_{IC}\frac{{C}_{1}\,{I}_{1}}{{s}_{I}+{I}_{1}}}}\limits_{\mathop{{\rm{stimulation}}}\limits_{{\rm{by}}\,{\rm{IL}}-2}}\mathop{\underbrace{-{m}_{C}\,{C}_{1}}}\limits_{{\rm{migration}}}\mathop{\underbrace{-{k}_{RC}\,{R}_{1}\,{C}_{1}}}\limits_{{\rm{inhibition}}\,{\rm{by}}\,{\rm{Treg}}}\mathop{\underbrace{-{\mu }_{C}\,{C}_{1}}}\limits_{{\rm{death}}}\mathrm{}.$$

CTLs are described by the Eq. (12). The action of ${D}_{f}$ cells activates CTLs, as defined by the “activaton by ${D}_{f}$” term, and IL-2 promotes their proliferation, as described in the “stimulation by IL-2” term. CTLs have two possible fates, either reduced by the “inhibition by Treg” and “death” terms, or released from the lymphoid tissue compartment, as described by the “migration” term.

13 $$\frac{d{R}_{1}}{dt}=\mathop{\underbrace{{a}_{DrR}\,{D}_{r}}}\limits_{\mathop{{\rm{production}}}\limits_{{\rm{by}}\,{{\rm{D}}}_{{\rm{r}}}}}\mathop{\underbrace{+{a}_{IR}\,{I}_{1}}}\limits_{\mathop{{\rm{stimulation}}}\limits_{{\rm{by}}\,{\rm{IL}} \mbox{-} 2}}\mathop{\underbrace{-{m}_{R}\,{R}_{1}}}\limits_{{\rm{migration}}}\mathop{\underbrace{-{\mu }_{R}\,{R}_{1}}}\limits_{{\rm{death}}}-\mathop{\underbrace{{k}_{antiRR}\,ant{i}_{R}\,{R}_{1}}}\limits_{{\rm{anti}} \mbox{-} {\rm{Tregdrug}}}\mathrm{}.$$

The Eq. (13) represents the Treg dynamics. ${D}_{r}$ induces Treg differentiation, as defined by the “production by ${D}_{r}$” term, and IL-2 promotes their proliferation, as described in the “stimulation by IL-2” term. Tregs are either released from the lymphoid tissue, or induced to apoptosis by the “death” term and by the “anti-Treg drug” term, if administered ($ant{i}_{R}=1$).

14 $$\frac{d{I}_{1}}{dt}=\mathop{\underbrace{{a}_{CI}\,{C}_{1}}}\limits_{\mathop{{\rm{production}}}\limits_{{\rm{by}}\,{\rm{CTL}}}}\mathop{\underbrace{-{\mu }_{I}\,{I}_{1}}}\limits_{{\rm{death}}}\mathop{\underbrace{-{k}_{antiI}\,ant{i}_{I}\,{I}_{1}}}\limits_{{\rm{anti}} \mbox{-} {\rm{IL}}-2\,{\rm{drug}}}\mathrm{}.$$

Equation (14) defines IL-2 dynamics within the lymphoid tissue, similarly to Eq. (8) in the prostate gland compartment. Taking into account that CTLs and IL-2 are tissue-associated, all terms have been previously described.

#### Treatments

The model provides a description of seven treatments (Table 1), two of which are not described by a dedicated equation. In detail, the administration of androgen deprivation therapy is implemented by the term −${\lambda }_{A}{1}_{CX}$ in Eq. (9), while the injection of NK cells is simulated by changing the initial value of NK variable (see Materials and methods).

The other five treatments have been modeled using the following ODEs to represent the treatment decays:

15 $$\frac{dV}{dt}=-\,{\lambda }_{V}\,V;$$

16 $$\frac{dant{i}_{I}}{dt}=-\,{\lambda }_{AI}\,ant{i}_{I};$$

17 $$\frac{dant{i}_{R}}{dt}=-\,{\lambda }_{AR}\,ant{i}_{R};$$

18 $$\frac{dant{i}_{M}}{dt}=-\,{\lambda }_{AM}\,ant{i}_{M};$$

19 $$\frac{d{I}_{CB}}{dt}=-\,{\lambda }_{ICB}\,{I}_{CB}\mathrm{}.$$

The Eqs. (15), (16) and (17) have been presented in Peng *et al*.^46^ and describe the dynamics of vaccine, anti-IL-2 and anti-Treg drugs, respectively. In our model, we introduce the decay of the anti-MDSC drug, described by Eq. (18), and the degradation of the ICB drugs as defined by Eq. (19). Each degradation rate has been calculated as explained in Eq. (9) description, considering the following half-lives:

- ${t}_{1/2}^{V}=7$ days (as in^46^);
- ${t}_{1/2}^{AI}=7$ days (as in^46^);
- ${t}_{1/2}^{AR}=7$ days (as in^46^);
- $${t}_{1/2}^{AM}=3.5$$ hours^71^;
- $${t}_{1/2}^{ICB}=5$$ days^72,73^.

### Model simulations and sensitivity analysis

**Figure 2:**
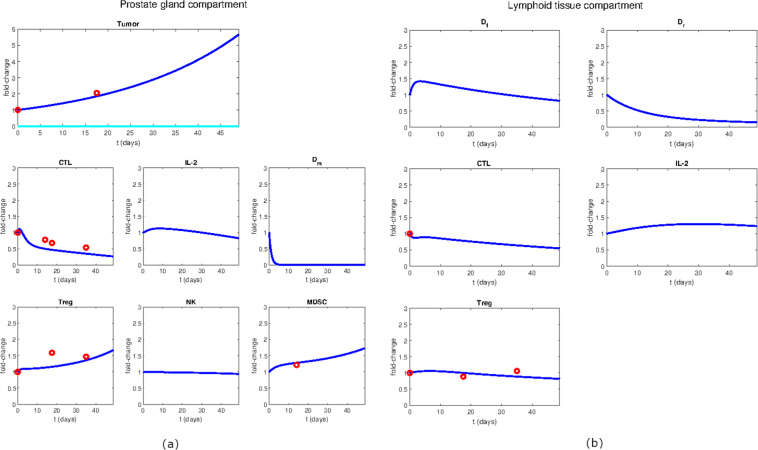
Model dynamics of the untreated case. (**a**) Variables of the prostate gland compartment. (**b**) Variables of the lymphoid tissue compartment. The simulation time is 7 weeks (49 days). The variable dynamics are expressed in terms of fold-change with respect to their initial values. The experimental data (red dots) are compared to the simulated behaviors (solid lines). Within the tumor chart, the dark blue line represents the total tumor volume while the light blue line represents the predicted AIPC dynamics.

**Figure 3:**
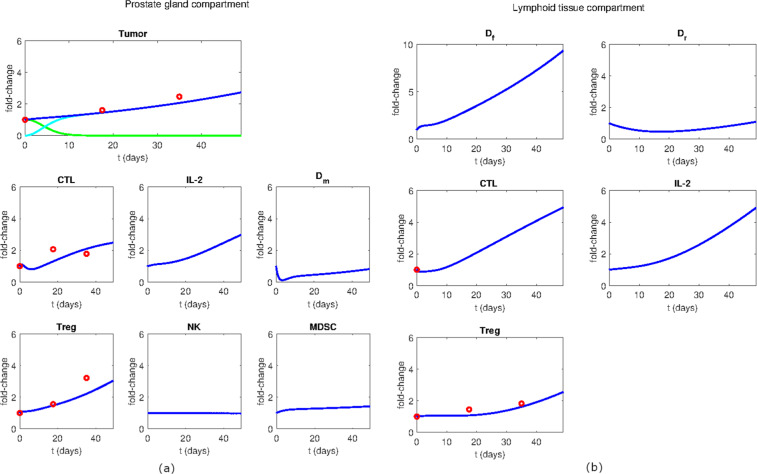
Model dynamics of the androgen deprivation treatment. (**a**) Variables of the prostate gland compartment. (**b**) Variables of the lymphoid tissue compartment. The simulation time is 7 weeks (49 days). The variable dynamics are expressed in terms of fold-change with respect to their initial values. The experimental data (red dots) are compared to the simulated behaviors (solid lines). Within the tumor chart, the dark blue line represents the total tumor volume while green and the light blue lines represent the predicted ADPC and AIPC dynamics, respectively.

We next verified if the model herein developed could capture the effect of a variety of experimentally tested treatments. To keep the simulation consistent with the experimental data, the system was simulated for 7 weeks (49 days), starting from an already developed cancer. In fact, this is the longest period considered in the *in vivo* experiments carried out to generate the data included in our model (see Materials and Methods for a complete description of the experimental data taken into account for model calibration). Overall, the model is in agreement with the considered data; it captures the temporal growth of the tumor volume, as well as the modulation of immune cell levels. In this section, the dynamics of the main system variables in the untreated scenario (Fig. 2) and after androgen deprivation therapy (Fig. 3) are described. While the untreated case represents the reference for evaluating the treatment effectiveness, understanding the behaviour of the system after androgen deprivation is crucial because this is the first-line treatment in advanced hormone-sensitive prostate cancers^40^, and in the clinical practice it is currently administered before immunotherapy. In the absence of any treatments, the model simulation (Fig. 2) shows that the tumor expands up to six times its initial volume before reaching the endpoint (dark blue line in the tumor chart). In parallel, both Tregs and MDSCs increase almost twice within the 7 weeks. Conversely, the number of CTLs and mature dendritic cells decreases over time, showing a progressive establishment of an immunosuppressive tumor microenvironment. It is noteworthy that, in the absence of androgen deprivation therapy, the entire tumor (dark blue line) is predicted to be androgen dependent.

When the model is simulated with the androgen deprivation therapy, the tumor evolves to its AIPC form, as shown in Fig. 3. In this scenario, the total tumor (dark blue line in the tumor chart) is growing at a slower pace than in the untreated simulation and, by the end of the simulation (7-week time point), it reaches a three-times smaller volume than in the untreated case. In the prostate gland compartment, we observed a marked increase in CTLs and Tregs, while mature dendritic cells showed a less pronounced growth. Unlike the untreated case, the androgen deprivation therapy induces an increase in the amount of dendritic cells as well as cytotoxic and regulatory T cells in the lymphoid tissue compartment.

We also considered data derived from experiments including the following immunotherapies: anti IL-2, anti-Treg, vaccine, NK administration, ICB and anti-MDSC (Supplementary Figs. S1–S9). The simulations of these scenarios show that androgen deprivation therapy is highly potentiated by ICB and anti-MDSC, in agreement with the experimental data^51^. In particular, our model predicts that the therapeutic scheme in which androgen deprivation is followed by a combination of ICB and anti-MDSC diminishes the tumor size by 30% at 7-week time point, a reduction greater than 70% compared to the untreated case (Fig. S8).

To evaluate the reliability of model predictions with respect to the uncertainty of parameter estimates, we performed a local and a global sensitivity analysis of the tumor variable defined as the sum of the ADPC and AIPC. The Local Sensitivity Analysis (LSA) highlights the obvious high dependence of tumor on its death and proliferation rates in all the reproduced experimental scenarios (see Supplementary File 3). Moreover, when the ICB treatment is administered, also the CTL activity influences the tumor growth as well as the maximal effect of ICB drug ${\delta }_{ICB}$, as expected. These results have been also confirmed by the Global Sensitivity Analysis (GSA), which predicts a high dependence of tumor on its proliferation and death rates, as well as on those parameters which regulates the killing abilities of the immune system, such as the maximal killing rate of tumor by CTLs and NK cells (${k}_{CX}$ and ${k}_{NT}$, respectively, see Supplementary File 4). Moreover, also the parameters describing the action of Treg, MDSC and ICB are predicted to influence the tumor growth, especially when the corresponding therapies are administered. For example, in case of ICB therapy, the perturbation of the parameter ${\delta }_{ICB}$, describing the effect of ICB drug on the CTL tumor killing rate, induces a strong variation in tumor size. The results of the LSA and the GSA suggest that the tumor dynamics is not significantly affected by parameter uncertainty, except for the few parameters that we expect playing a crucial role. For technical details see Materials and Methods.

### Identification of effective drug combinations for prostate cancer treatment

After having verified the capability of our model to reproduce the experimental data, we explored the possibility of using the model to predict the efficacy of the combination therapies. To compare all the treatment combinations, we defined a standard in silico protocol of drug administration. In these simulations, all treatments started simultaneously at week 0 and ended at week 4.

**Figure 4:**
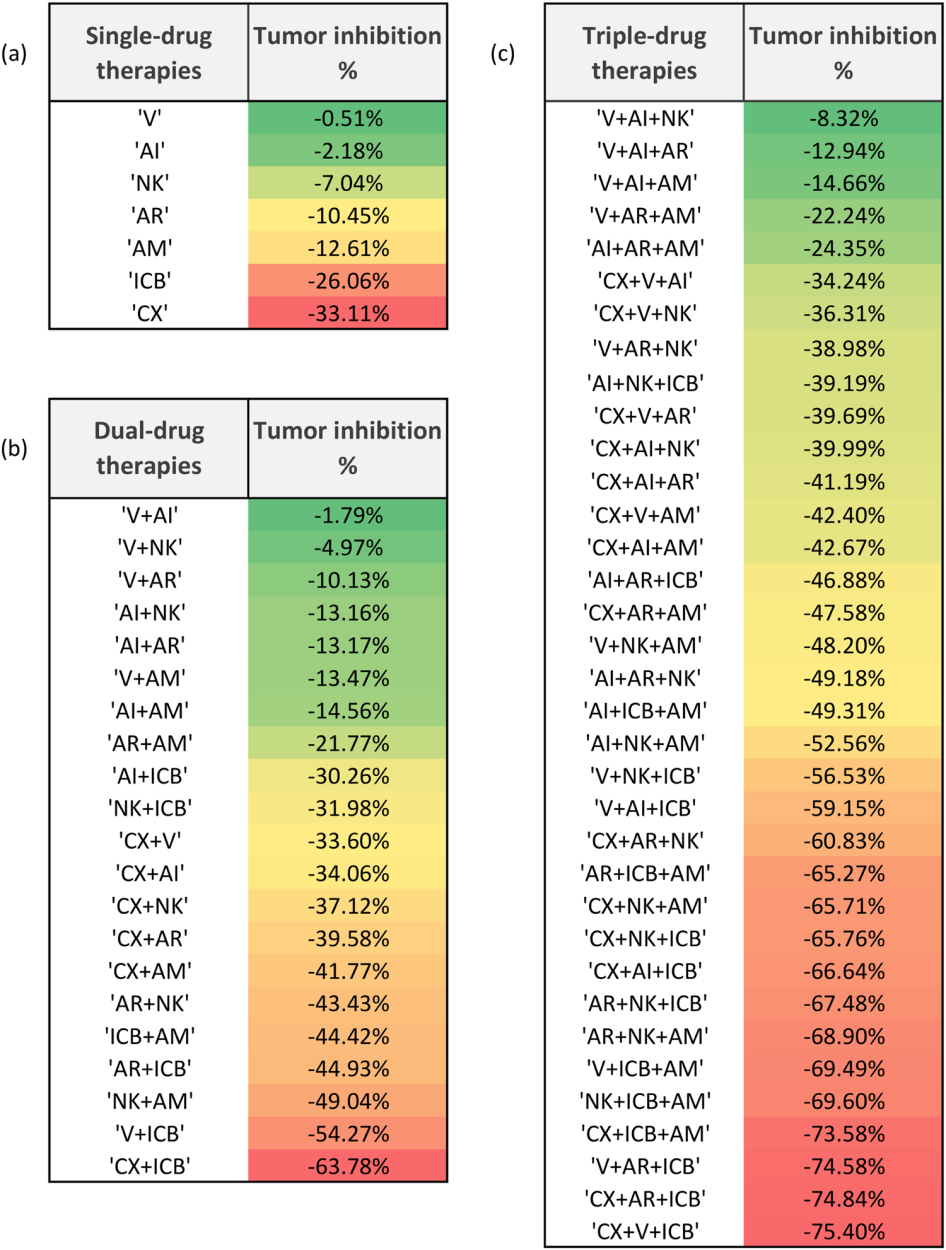
Therapy effect on tumor inhibition. The model-predicted effect of (**a**) single-, (**b**) dual- and (**c**) triple-drug therapies. Therapies have been sorted by their tumor inhibition percentage compared to the untreated case, after $4$ weeks of therapy. Treatments are named by the following abbreviations: Androgen Deprivation (CX), Anti-IL-2 (AI), Anti-Treg (AR), Anti-MDSC (AM), Vaccine (V), injection of NK cells (NK) and Immune-Checkpoint Blockade (ICB).

The efficacy of the therapy was evaluated comparing the predicted tumor size at week $4$ of therapy simulation with the tumor size in the untreated scenario at the same time point. A summary of the most and least successful therapies, divided by the number of included treatments, is reported in Table 2 and a graphical representation of the results for single-, dual- and triple-drug therapies is visualized in Fig. 4.

**Table 2:** Table of the model predicted best and worst treatment protocols.

Number of combined treatments	Best combination	Worst combination
2	CX + ICB	V + AI
3	CX + V + ICB	V + AI + NK
4	V + AR + ICB + AM	V + AI + AR + AM
5	V + AR + NK + ICB + AM	CX + V + AI + AR + AM
6	CX + V + AR + NK + ICB + AM	CX + V + AI + AR + NK + AM

The most and the least efficacious combination therapies sorted by the total number of treatments included in each therapy. Therapies are indicated by the following abbreviations: Androgen Deprivation (CX), Anti-IL-2 (AI), Anti-Treg (AR), Anti-MDSC (AM), Vaccine (V), infusion of NK cells (NK) and Immune-Checkpoint Blockade (ICB).

When monotherapy is considered, our simulations indicate that castration is the the most effective treatment, with a 33% smaller tumor size at week 4 compared to the untreated. On the other hand, cancer vaccination is predicted to be the least beneficial one, with almost no effect on the tumor (Fig. 4(a)). In the case of dual-drug therapies, androgen deprivation combined with one immunotherapy is shown to increase the percentage of tumor reduction in all cases (Fig. 4(b)). In particular, androgen deprivation coupled with ICB is predicted to be the most effective therapy, with 64% tumor reduction compared with the untreated. Conversely, the addition of vaccination to androgen deprivation induces a less than one percent decrease, suggesting a minimal contribution of this immunotherapy to contain the tumor burden. However, vaccination is showing a complex behaviour and its contribution to the tumor-size reduction is highly dependent on the other drugs included in the combination therapy. For example, it shows an antagonistic behaviour with anti-IL2, anti-Treg and NK infusion, because the predicted tumor inhibition of these treatments, tested as single drugs, is higher than the one obtained by combining them with the vaccine. Conversely, vaccine exhibits an additive effect when combined with androgen deprivation or anti-MDSC and a synergistic effect with ICB (Fig. 4(b)). Furthermore, when vaccine is administered in combination with androgen deprivation and ICB, we observed a 75% tumor reduction, reaching the highest percentage of tumor reduction among the triple-drug therapies (Fig. 4(c)). Additional simulations performed combining four, five, and six treatments are shown in Supplementary Fig. S12.

To further explore the presence of synergistic effects among the treatments, we computed the Bliss Combination Index (BCI)^74,75^, a commonly used method to assess the combined effect of drugs. The BCI of two treatments A and B is calculated as

20 $$BCI(A,B)=\frac{{T}_{i}(A\cup B)}{{T}_{i}(A+B)}=\frac{{T}_{i}(A)+{T}_{i}(B)-{T}_{i}(A){T}_{i}(B)}{{T}_{i}(A+B)},$$

where ${T}_{i}(x)$ indicates the predicted percentage of tumor inhibition obtained by applying treatment $x$ compared to the untreated case. In ${T}_{i}(A+B)$, the therapy effect comes from the interaction of treatments $A$ and $B$ combined, while ${T}_{i}(A\cup B)$ represents the effect of $A$ and of $B$, as two non-interacting treatments (further details in Materials and methods). When $BCI(A,B\mathrm{)\;  < \; 1}$, the two treatments are predicted to be synergistic because the predicted effect on tumor size of the combination therapy $A+B$ is stronger than the effect of the two treatments $A$ and $B$ separately. Conversely, when $BCI(A,B\mathrm{)\;  > \; 1}$, the two treatments are considered antagonists.

**Figure 5:**
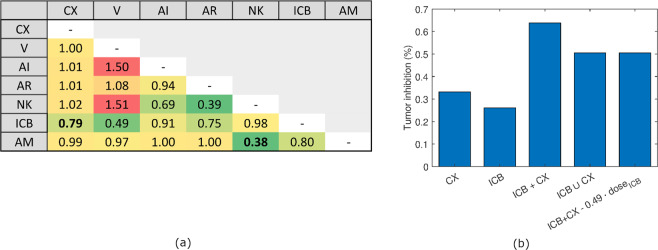
Synergistic treatments. (**a**) Treatment BCIs calculated between the corresponding column and row label. BCIs of the most synergistic treatments among all (NK + AM) and the most synergistic treatments relative to androgen deprivation (CX + ICB) have been highlighted in bold. (**b**) Tumor inhibition rate of CX and ICB as monotherapies, of ICB and CX interacting as a dual-therapy, of ICB and CX not interacting as they were delivered separately, and of ICB half-dosage and CX interacting as a dual-therapy.

Considering dual-drug therapies, this analysis identified anti-MDSC and NK infusion as the most synergistic treatments (BCI = 0.38), as shown in Fig. 5(a). Despite being highly synergistic, this therapy does not reach the highest control of tumor size, that is, instead, obtained coupling CX and ICB, as described above. Indeed, the BCI of CX + ICB is 0.79, indicating a modest synergy that is nonetheless sufficient to reach a 64% tumor inhibition (Fig. 4(b)). In addition to the dual-drug therapies, the BCI can be computed for all treatment combinations, as reported in Supplementary File 5. By considering all the possible combinations, the most synergistic therapy is given by the the addition of AM to a therapy that includes V and NK infusion.

We also attempted to use these results to estimate the minimal dose to maintain a satisfactory treatment efficacy. For this analysis, the minimal satisfactory tumor inhibition was fixed to the theoretical value obtained assuming that the two drugs have no interaction ($A\cup {\rm{B}}$ in Eq. (20)). If we consider the most promising dual-drug therapy, CX and ICB, half-dose of ICB is enough to obtain the same effect of $ICB\cup CX$ (Fig. 5(b)).

**Figure 6:**
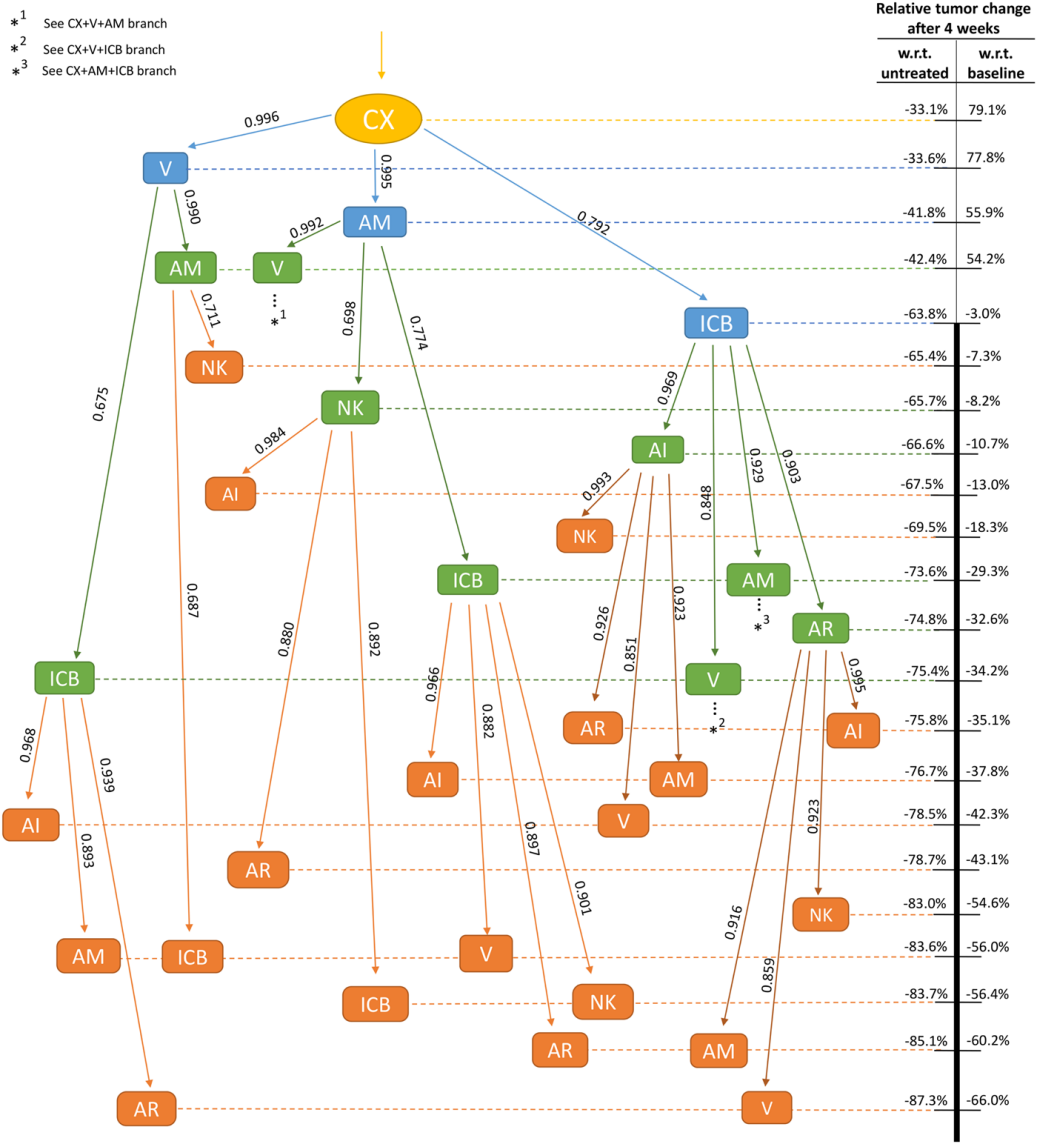
Treatment decision tree. Limiting the tree to androgen deprivation as root node, therapies have been step-wise combined to synergise (BCI reported on the corresponding edge). Each node position has been computed according to the relative tumor change after 4 weeks (scale on the right). Combined therapies at the bottom are the most effective on the tumor size. The scale thickness emphasizes the nodes able to decrease the tumor size with respect to (w.r.t.) the baseline. For simplicity, branches showing the same pattern have been replaced by stars indicating the branch of reference (${\ast }^{1}$ = CX + V + AM, ${\ast }^{2}$ = CX + V + ICB, ${\ast }^{3}$ = CX + AM + ICB). Treatments are named by the following abbreviations: Androgen Deprivation (CX), Anti-IL-2 (AI), Anti-Treg (AR), Anti-MDSC (AM), Vaccine (V), injection of NK cells (NK) and Immune-Checkpoint Blockade (ICB).

The results obtained from synergy analysis and tumor inhibition evaluation are complementary and their combination is essential for a more complete view of the system behavior. Hence, we built a decision tree that integrates the computed synergistic effect and the predicted percentage of tumor inhibition (Fig. 6). Motivated by the lack of efficacious treatments for advanced castration resistant PCa, we set out to identify an effective immunotherapy for subjects with AIPC, which in our model develops after androgen deprivation therapy. For this reason, the androgen deprivation therapy has been set as the root of the decision tree. The immunotherapies have been included as internal nodes, connected to each other and to the root by edges, annotated with the BCI value. Only edges with a synergistic value (BCI <= 0) are reported. The paths from the root to the terminal nodes represent the possible combined therapies and the position of the nodes along the tree reflects the efficacy of the therapies in inhibiting the tumor growth, according to the scale reported on the right (the most effective therapies are positioned lower down). This chart highlights that adding ICB to CX seems the most promising therapeutic combination to keep under control the tumor growth, notwithstanding the need to add at least one additional treatment to reduce more than 3% the tumor size compared to the baseline. If additional therapies are added, our simulations suggest vaccination as the most effective, leading to a 34% tumor reduction compared to baseline and 75% compared to the untreated.

## Discussion

In this study, we developed a new mathematical model of PCa to predict effective combination immunotherapies. Compared with the existing PCa models^46,59,76–81^, we included more mechanistic details about the tumor microenvironment. These extensions allowed us to test different types of immunotherapy and obtain a comprehensive view of the treatment effects. In this model, we implemented the immuno-suppression exerted by MDSCs, immune cells that have been recently demonstrated to be important contributors to PCa progression^50,51,82–85^. Targeting MDSCs is emerging as an attractive therapeutic option to improve the response rate to immunotherapy and patient survival in several cancers^86^. Interestingly, a recent phase II clinical trial in melanoma (NCT02403778) showed that the addition of an anti-MDSC agent to ICB therapy seems to be safe and it increases the number of activated T cells^87^. However, additional clinical studies are needed to evaluate the effectiveness of this approach. Furthermore, we added NK cells, components of the innate immune branch exerting a cytotoxic action without the need of prior antigen exposure^88,89^. The potential of NK cells in cancer immunotherapy has been pointed out by numerous preclinical studies that showed several attractive features of these cells^90^. Above all, NK cells display an overall safe profile, given by their limited *in vivo* persistence, the lack of clonal expansion, and the absence of the immune rejection associated with allogeneic transplantation^91^. Despite these positive characteristics, the use of NK cell-based immunotherapy in clinical settings is still at the beginning and the availability of a mathematical model describing NK cell action could support the design of new studies. In addition, we included the ICB treatment as an increment in the CTL tumor-killing capacity, according to the biological evidences indicating that immune-checkpoints limit T cell effector functions^54,92,93^. The ICB therapies have been proven promising for several types of solid tumors, leading an increasing interest also in the context of prostate cancer^94^. In recent years, the FDA approved ICB treatments for some tumors, such as metastatic melanoma and renal carcinoma^95^, and a combination of two different ICB drugs is currently under phase II clinical trial for metastatic castration-resistant prostate cancer^96^ (NCT02985957).

Our model offers the possibility of testing *in silico* a variety of combinatorial immunotherapies and identifying the most promising ones. In particular, in this study we evaluated the effect of combination immunotherapies in subjects developing AIPC after androgen deprivation therapy, considering both the impact on tumor size and the synergistic effects. Given the high number of immunotherapies currently available, it is not feasible to test all combinations in animal models and clinical setting. Our mathematical model offers a cost-effective approach to identify *in silico* the most promising combination therapies. This is particularly relevant for prostate cancer, a type of tumor that so far did not respond well to immunotherapy. The ICB therapy, for example, despite the promising results in the treatment of other solid tumors, has not yet been approved for prostate cancer. A possible explanation of the lack of satisfactory results could be due to a strong immuno-suppressive tumor microenvironment^44,97,98^. Interestingly, our simulations indicate that anti-MDSC combined with ICB almost double the efficacy of ICB alone (44% vs 26% tumor reduction compared to untreated). When considering triple-drug therapies, our results showed ICB combined with vaccine as the most effective dual-drug immunotherapy in subjects under androgen deprivation therapy. Interestingly, a phase II clinical trial testing androgen deprivation therapy and ICB reported a reduction in PSA levels and tumor reduction in 25% of patients^99^ and a phase I clinical trial of the PCa vaccine sipuleucel-T combined with ICB gave promising results. The therapy was well tolerated and a significant increase in serum antibodies specific for the prostate tumor-associated antigen was observed^100^.

In conclusion, the mathematical model presented here serves as a tool to test *in silico* the efficacy of cancer immunotherapies. To bypass the paucity of public data from human studies, the model has currently been calibrated using data derived from prostate cancer mouse models and *in vitro* experiments. To investigate the impact of data availability on model predictions, we performed a local and a global sensitivity analysis on the tumor size, which is the main variable discussed in the result section (see Materials and Methods, Supplementary Files 3 and 4). As expected, both the local and global sensitivities confirmed that the tumor dynamics is predicted to be highly influenced only by its proliferation and death rates as well as other parameters strictly connected with the therapy administered. These results suggest that the model predicted tumor size is poorly affected by parameter uncertainty, except for the few parameters playing a crucial role in tumor dynamics. Despite this result provides an additional indication of the reliability of model predictions, additional efforts could be devoted to identify a new calibration targeted to human data. In this case, it would be fundamental to add a peripheral compartment to the model, because most of the human data derives from peripheral tissues, such as blood. In this context, our model represents an initial step toward the development of a QSP model including the pharmacokinetics and pharmacodynamics descriptions.

Future extensions could also address the mechanism of action of the drugs. For example, we could better describe MDSC and Treg regulations, considering the intracellular signaling of these cells to identify new targets of anti-MDSC and anti-Treg drugs. A more detailed description of the cytokine dynamics could be performed by introducing other immune cells responsible for IL-2 production, such as T helper cells, which have been mathematically described, even if not in the context of prostate cancer^68,101^. Moreover, we could improve the representation of the tumor microenviroment, by introducing the Transforming Growth Factor Beta (TGF-$\beta$), which has been recognized to be important in several tumor progressions^102^. The importance of TGF-$\beta$ has been confirmed for prostate cancer^103^ and it could be a potential target of prostate cancer therapies. The role of TGF-$\beta$ has been investigated also by means of a mathematical model based on partial differential equations^104^. The model capabilities could also be enhanced by including in the tumor equations a stochastic “mutation” term, which describes the evolution from ADPC into AIPC considering both the mutation randomness and the selective pressure due to androgen deprivation therapy. An additional aspect that could be taken into account is the toxicity of the treatments. For example, some immunotherapies, by enhancing T cell activity, can induce strong, life-threatening immune reactions^105^. In this perspective, the availability of data related to the treatment side-effects would be highly beneficial because it would allow extending the synergy analysis by including the toxicity effect^106^ and thus better supporting clinical decision-making.

## Materials and methods

### Computational environment

The model has been implemented as a set of Matlab functions, which have been simulated by the state-of-the-art *ode15s* integrator. For the model calibration, we used the evolutionary strategy provided in the optimization toolbox of Matlab: the genetic algorithm (*ga*). The optimization algorithm has been parameterized considering a population size of 200 individuals, a crossover fraction of 0.9 and a tolerance function of 10^−2^.

### Experimental data

Model calibration has been performed taking into account a wide range of published data^31,46,49–51^.

For consistency purposes, our model includes data presented by Peng *et al*.^46^. Tumor size and populations of CTLs and Tregs in prostate gland and lymphoid tissues of prostate-specific Pten -/- mice were measured every 2.5 weeks for 5 weeks, starting after 14 weeks of mouse-life, when the developed prostate cancer reaches a volume large enought to be treated. Pten -/- mouse model has been specifically selected to mimic the natural tumorigenesis of human prostate cancer development as well as the effects of treatment^107^.

CTLs and MDSCs infiltrating prostate cancer have been counted as ratios at week 6, 9 and 16 of life of untreated prostate-specific Pten -/- mice by Garcia *et al*.^50^ and the presence of MDSCs in the tumor microenvironment has been proven to reduce CTL’s concentration of 42% *in vitro*.

Two *in vitro* human prostate cancer cell lines co-cultured with NK cells provided data of NK-dependent tumor regression^49^, halving the tumor size within 48 hours.

The optimal NK injection dose has been calculated following Domogala *et al*.^108^ indications.

The effects of androgen deprivation combined to either ICB or anti-MDSC alone or the two treatments together have been measured delivering the androgen deprivation to 14 weeks-old severily-mutated Pten mice and the immunotherapy 3 weeks later^51^. The anti-MDSC and ICB immunotherapies were delivered 3 times per week and everyday, respectively, for 4 weeks before endpoint analysis.

Specifically, the ICB treatment is defined by a cocktail of anti-CTLA4 and anti-PD1 antibodies, while the anti-MDSC treatment has been performed by Cabozatinib alone. Specifically, the ICB treatment is defined by a cocktail of anti-CTLA4 and anti-PD1 antibodies, while the anti-MDSC treatment has been performed by Cabozatinib alone.

### Model calibration

The model comprehends $51$ parameters, but $23$ of these are directly taken from the literature or computed as specified in the main text (e.g. Equation (5)) without relying on numerical optimization methods. The remaining $28$ parameters have been numerically estimated. Among these, $18$ have been only refined by the optimization function in an interval close to their literature values, while the other $10$ parameters have been entirely estimated in this work. For a detailed description of the estimation procedure, we refer to Supplementary Table S1.

The optimization function that we created to estimate the parameters is composed of the sum of three main parts that correspond to the experimental scenarios discussed in Experimental data section:

$$Opt=Op{t}_{1}+Op{t}_{2}+Op{t}_{3}.$$

To extrapolate CTL and MDSC cell counts of 14 weeks-old mice (simulation starting point), we interpolated Garcia’s experimental data^50^ by linear regression. To properly adapt Garcia’s quantitative data to Peng’s relative data, we calculated the ratio between cell counts at 16 and 14 weeks. We integrated the resulting relative data to Peng’s experimental data to define the function $Op{t}_{1}$. We fitted the model by minimizing the absolute difference between model-predicted variable dynamics and experimental data:

$$Op{t}_{1}=\frac{1}{7}\,\mathop{\sum }\limits_{i=1}^{7}\,\left[\frac{1}{{M}_{i}}\,\mathop{\sum }\limits_{j=1}^{{M}_{i}}\,\left(\frac{1}{{N}_{j}}\,\mathop{\sum }\limits_{k=1}^{{N}_{j}}\,|{X}_{k}-{\overline{X}}_{k}|\right)\right],$$

where 7 is the number of combination therapies considered in Peng *et al*.^46^, for each combination therapy ${M}_{i}$ is the number of variables requiring data extrapolation, for each variable ${N}_{j}$ is the number of time-point dependent data, ${X}_{k}$ is the estimated variable value computed by simulating the model and ${\overline{X}}_{k}$ is the corresponding experimental data.

The second term $Op{t}_{2}$ takes into account the *in vitro* human prostate cancer cell lines from Lin *et al*.^49^ and the *in vitro* Pten null prostate cancer cell lines from Garcia *et al*.^50^. The initial values of all model variables not present in the experiments of interest have been set to zero. The initial values of NK and PCa have been reported in Wang *et al*.^31^, while CTL and MDSC have been extrapolated from Garcia’s experimental data^50^ as previously described. We used a weighted least squares method to fit the experimental data:

$$Op{t}_{2}={w}_{1}{\left(\frac{{T}_{NK}}{T}-0.5\right)}^{2}+{w}_{2}{\left(\frac{{C}_{M}}{C}-0.42\right)}^{2},$$

where ${w}_{1}$ and ${w}_{2}$ are the empirically estimated weights required to consistently scale the value of $O{p}_{2}$ with respect to the other two components $Op{t}_{1}$ and $Op{t}_{3}$. ${T}_{NK}$ is the model-predicted tumor amount co-cultured with NK cells for 2 days, $T$ is the model-predicted tumor amount cultured without NK cells, ${C}_{M}$ is the model-predicted number of CTLs co-cultured with MDSCs for 5 days, while $C$ is the model-predicted count of CTLs cultured without MDSCs. The percentages 0.5 and 0.42 derive from experimental data.

Following a similar strategy, we used data of ICB and anti-MDSC drugs to define the last part of the optimization function $Op{t}_{3}$. In our model, immunotherapies have been administered as in Lu *et al*.^51^ and the average measures of tumor size and MDSC population have been considered as effects of the immunotherapies. We compared the castration-resistant tumor size after 7 weeks of either ICB or anti-MDSC drugs alone or their combination versus the immunotherapy-free control. The same procedure has been repeated for MDSCs. The two ratios have been optimized through weighted least squares method:

$$Op{t}_{3}=\mathop{\sum }\limits_{k=1}^{3}\,{\hat{w}}_{k}\cdot {\left(\frac{{T}_{k}}{{T}_{cx}}-{\overline{Rt}}_{k}\right)}^{2}+\mathop{\sum }\limits_{k=1}^{3}\,{\tilde{w}}_{k}\cdot {\left(\frac{{M}_{k}}{{M}_{cx}}-{\overline{Rm}}_{k}\right)}^{2},$$

where the summations from 1 to 3 fit the three treatment conditions. ${\hat{w}}_{k}$, ${\tilde{w}}_{k}$ are the empirically estimated weights required to consistently scale the value of $O{p}_{3}$ with respect to the other two components $Op{t}_{1}$ and $Op{t}_{2}$. ${T}_{k}$ and ${M}_{k}$ represent the model-predicted amount of tumor and MDSCs, respectively, when the therapy $k$ is administered. These values are compared with ${T}_{cx}$ and ${M}_{cx}$ providing the model-predicted size of tumor and MDSC population under androgen deprivation alone. ${\overline{Rt}}_{k}$ and ${\overline{Rm}}_{k}$ derive from the experimental data^51^.

Considering the injected NK dosage tolerated in human as 9 times their physiological NK amount^108^, we increase NK initial value assuming the same multiplicative factor in mice

### Structural Identifiability analysis

A structural identifiability analysis has been performed by means of the Matlab toolbox GenSSI^53^. The Matlab sources needed to run the analysis are provided in the Supplementary File 2. The software requires: (i) a list of all the model variables and their relations inside the mathematical model; (ii) a list of all the model parameters, by also specifying the ones that have to be estimated; (iii) the model initial conditions for running the simulations; and (iv) a list of the experimentally observed quantities, which can be model variables or a function of these. Unfortunately, the algorithm has some limitations that prevent the complete specification of all the experimental data employed during the model calibration phase. In particular, we excluded from this analysis the *in vitro* data and the ratios of the system variables in different experimental conditions (see previous section for a complete description of the experimental data). Despite these limitations, the analysis guarantees a local identifiability.

### Local and global sensitivity analyses

A local and a global sensitivity analysis have been performed on tumor size, which is the main variable discussed in this work. To conduct the Local Sensitivity Analysis (LSA), we considered the total amount of tumor as $X={X}_{1}+{X}_{2}$ and we evaluated the tumor LSA value of a model parameter $p$ by using a logarithmic LSA^109^ as:

$${T}_{LSA}(p)=\frac{\delta \,\log (X(t,p))}{\delta \,\log (p)}=\frac{\delta X(t,p)\frac{1}{X(t,p)}}{\delta p\frac{1}{p}}=\frac{\delta X(t,p)}{\delta p}\frac{p}{X(t,p)}.$$

We approximated $\frac{\delta X(t,p)}{\delta p}$ by the central finite difference and we obtained:

$${T}_{LSA}(p)=\frac{X({t}_{f},p+\Delta p)-X({t}_{f},p-\varDelta p)}{2\cdot \Delta \cdot X({t}_{f},p)},$$

where ${t}_{f}$ is the last simulated time point (49 days). The results obtained by fixing $\Delta =1 \%$ are shown in Supplementary File 3.

For implementing the Global Sensitivity Analysis (GSA), we followed the standard approach consisting in evaluating the LSA for each model parameter, starting from random estimates generated inside the model parameter space, as described in the Zi *et al*. paper^110^. We sampled the space by generating 1000 random parameter estimates through Latin hypercube sampling^111^, and we defined the GSA for each model parameter as the median value of these 1000 LSAs.

To increase the reliability of the analysis, we computed the GSA by considering the complete LSA profiles:

$${T}_{LSA}(p)={\int }_{0}^{{t}_{f}}\,\frac{X(t,p+\Delta p)-X(t,p-\Delta p)}{2\cdot \Delta \cdot X(t,p)}\,dt,$$

while in the previous LSA we computed the tumor sensitivity only at the last simulated time point. This choice allowed to better capture the sensitivity of the overall tumor dynamics with respect to each parameter perturbation. The parameter space has been defined according to the following constraints. For the 28 parameters estimated by optimization methods, we used the same range of variability considered for the model calibration. For all the remaining parameters, we considered a variation of $\mathrm{30 \% }$ their initial literature estimate. The results of the GSA obtained by fixing $\Delta =1 \%$ are shown in Supplementary File 4.

### Bliss combination index

To evaluate the synergistic effects of the therapies we considered the Bliss combination index (BCI)^74^, which is one of the most commonly used correlation measures. BCI is defined as the ratio between the efficacy of two therapies supposing that these do not interact and the efficacy of the same therapies administered together. To calculate the BCI, we need to express treatment effects as probabilities. Then, the first term of the ratio is computed by assuming that each treatment is independent to the others. When this occurs, the effect of the two drugs considered alone is calculated by the formula:

$$P(A\cup B)=P(A)+P(B)-P(A\cap B),$$

that, considering the independence between A and B, becomes:

$$P(A\cup B)=P(A)+P(B)-P(A)P(B).$$

If we consider the tumor inhibition percentages as probabilities, we calculate the BCI of two treatments A and B as:

$$BCI(A,B)=\frac{{T}_{i}(A)+{T}_{i}(B)-{T}_{i}(A){T}_{i}(B)}{{T}_{i}(A,B)},$$

where ${T}_{i}(x)$ indicates the model-predicted percentage of tumor inhibition obtained by applying treatment $x$ with respect to the untreated case. In the case of ${T}_{i}(A,B)$, the treatment is given by the combination of the two treatments $A$ and $B$. When $BCI(A,B) < 1$, the model-predicted effect on tumor size of the combined therapy $A+B$ is stronger than the effect of the two therapies $A$ and $B$ alone. Therefore, the two therapies are predicted to be synergistic. Conversely, when $BCI(A,B) > 1$, the two therapies are considered antagonist.

## Supplementary information

Supplementary File 1.

Supplementary File 2.

Supplementary File 3.

Supplementary File 4.

Supplementary File 5.

Supplementary File 6.
