# Supplementary material for: A QSP model of prostate cancer immunotherapy to identify effective combination therapies
Source: Sci Rep. 2020 Jun 3;10:9063. doi: 10.1038/s41598-020-65590-0 (PMC7270132; doi:10.1038/s41598-020-65590-0)
Supplement: Supplementary file 1 [file 41598_2020_65590_MOESM1_ESM.pdf]

A QSP model of prostate cancer immunotherapy to identify effective combination therapies

Roberta Coletti [1,2], Lorena Leonardelli [2], Silvia Parolo[2] and Luca Marchetti[2,*]

[1] University of Trento, Department of mathematics, Trento, 38123, Italy
[2] Fondazione The Microsoft Research - University of Trento Centre for Computational and Systems Biology (COSBI), Rovereto, 38068, Italy
* Corresponding author: marchetti@cosbi.eu

Supplementary file 1.

Figures S1-S8 show the model dynamics for each experimental therapy[1–3] used to calibrate our model: S1 Vaccine, S2 androgen deprivation combined to anti-IL-2, S3 androgen deprivation combined to anti-Treg, S4 androgen deprivation combined to vaccine and anti-Treg, S4 androgen deprivation combined to vaccine, S6 androgen deprivation combined to ICB, S7 androgen deprivation combined to anti-MDSC, S6 androgen deprivation combined to ICB and anti-MDSC. Figure S9 shows the model dynamics after NK injection treatment, estimated from published *in vitro* data[1] as described in the Materials and methods section.

Figures S1-S9 are structured as follow: (a) variables of the prostate gland compartment, (b) variables of the lymphoid tissue compartment. All simulations start when Pten -/- mice are 14 weeks-old (simulation time zero) and run for 7 weeks (49 days), according to the longest administration protocol. The variable dynamics are expressed in terms of fold-change with respect to their initial values. The experimental data (red dots) are compared to the simulated behaviors (solid lines). Within the tumor chart, the dark blue line represents the total tumor volume while green and the light blue lines represent the predicted ADPC and AIPC dynamics, respectively. When androgen deprivation therapy is not administered, the AIPC does not evolve. NK injection, ICB and anti-MDSC treatments are not expressed as fold-changes between the baseline and the time-point of interest and, thus, the related experimental data are not shown in Figures S6-S9.

Figure S10 shows the change in castration-resistant tumor size (a) and in MDSC population (b) after either ICB or anti-MDSC single-therapies and after ICB and anti-MDSC dual-drug immunotherapy. Experimental data provided by Lu *et al.*[4].

Figure S11 shows (a) a 50% tumor size reduction after NK cell injection and (b) CTL population reduced by 42% after anti-MDSC treatment. Experimental *in vitro* data provided by Garcia *et al.*[3] and and Lu *et al.*[4] have been used to validate our model.

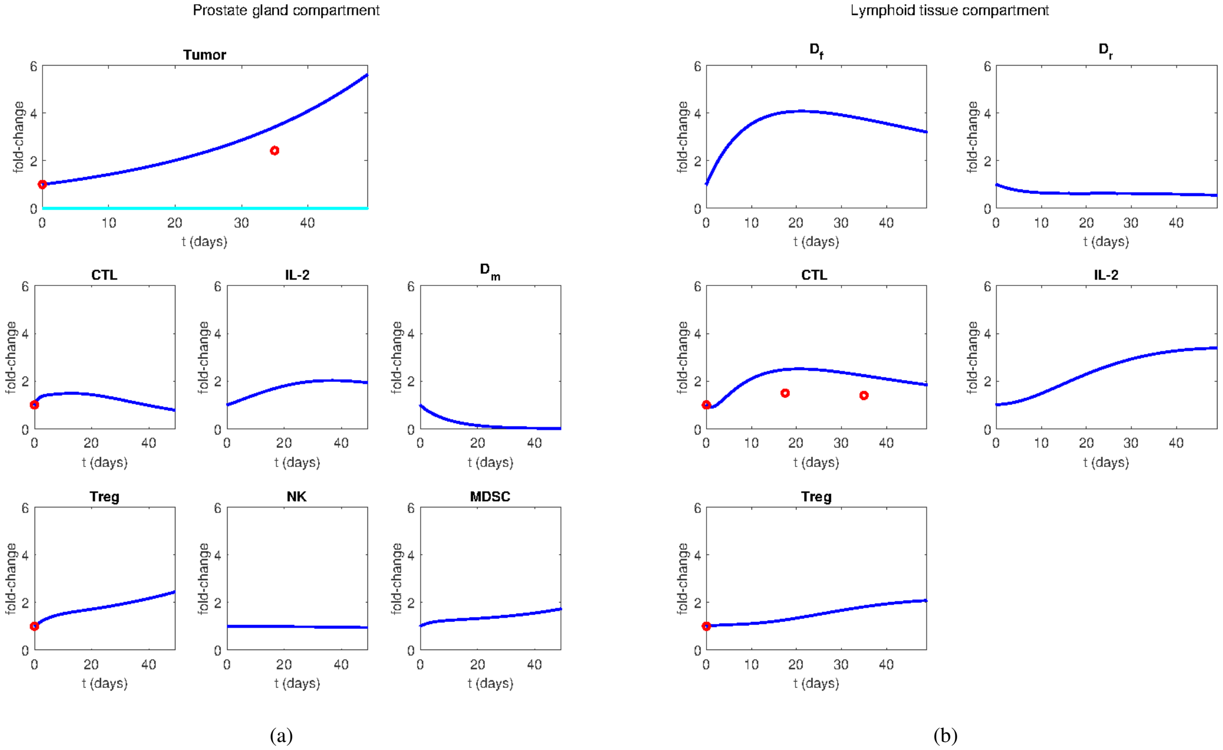

Figure S1. **Model dynamics of vaccine treatment.**

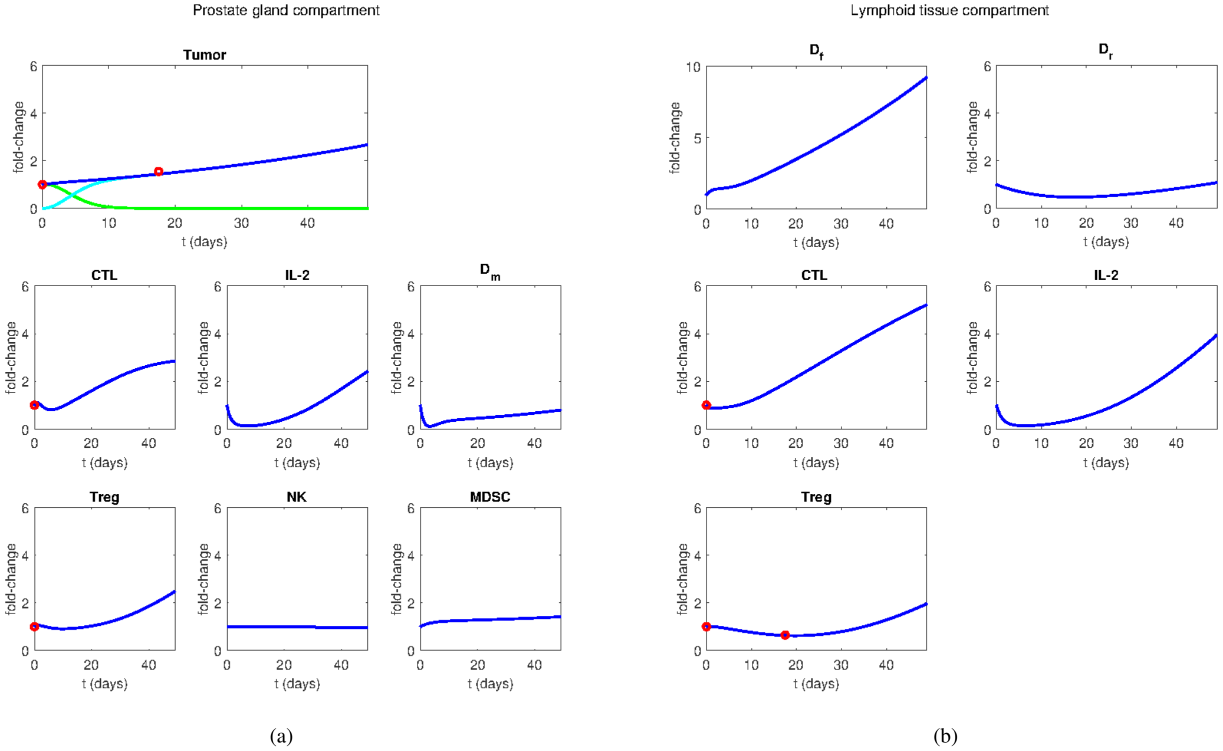

Figure S2. **Model dynamics of androgen deprivation combined to anti-IL-2 therapy.**

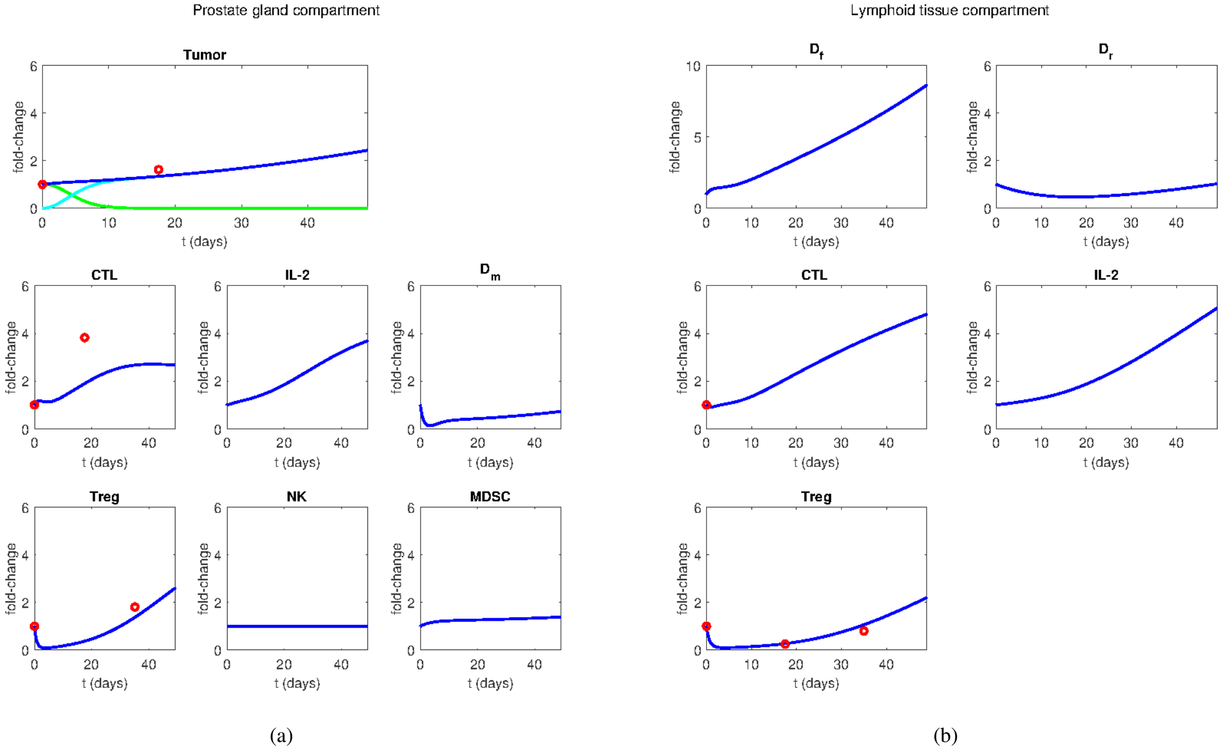

Figure S3. **Model dynamics of androgen deprivation combined to anti-Treg therapy.**

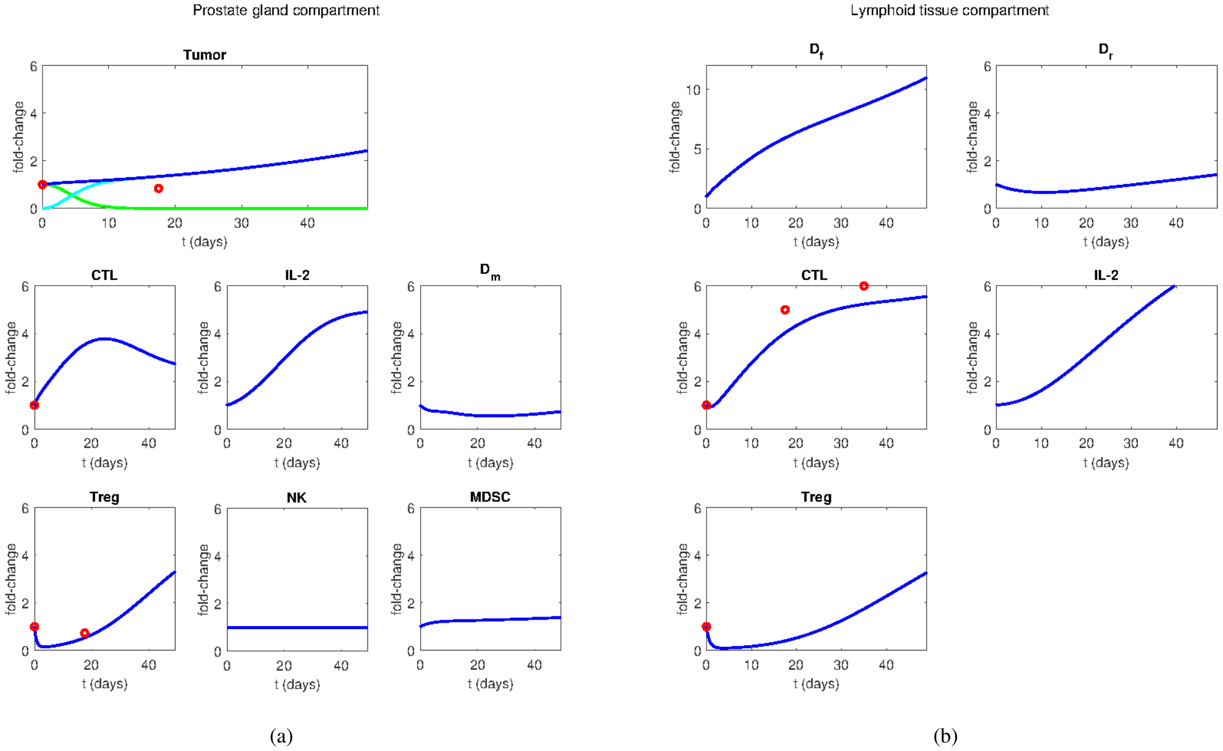

Figure S4. **Model dynamics of androgen deprivation combined to vaccine and anti-Treg therapy.**

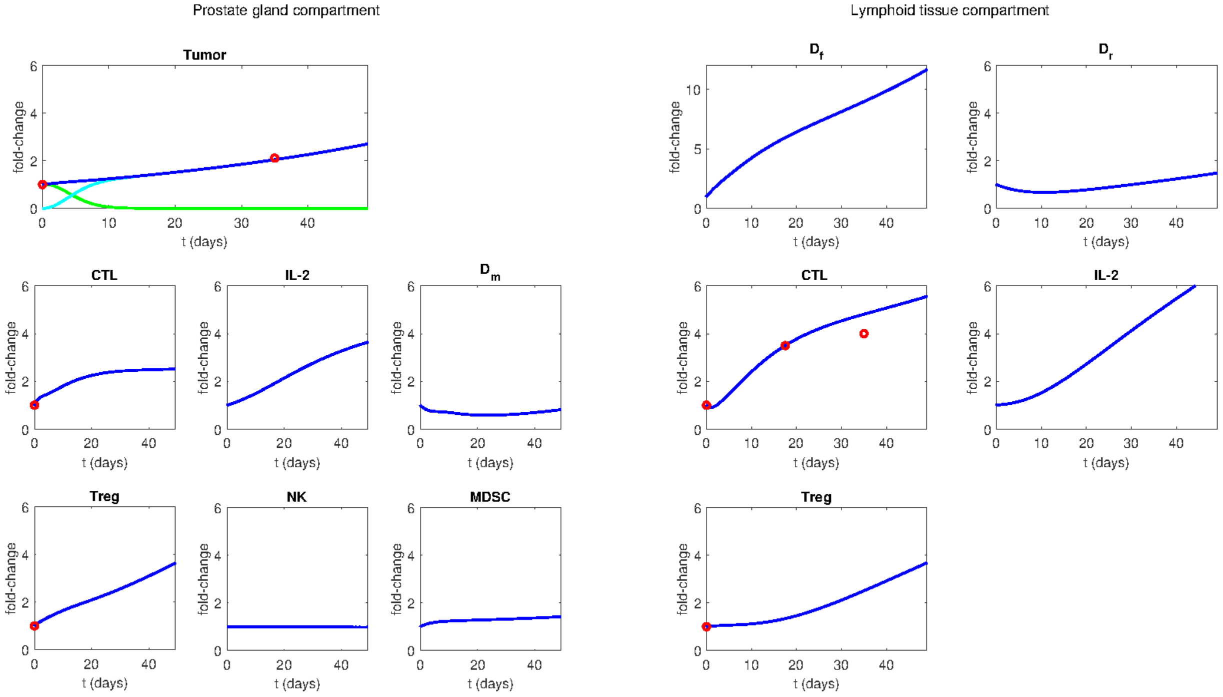

(a) (b)

Figure S5. **Model dynamics of androgen deprivation combined to vaccine therapy.**

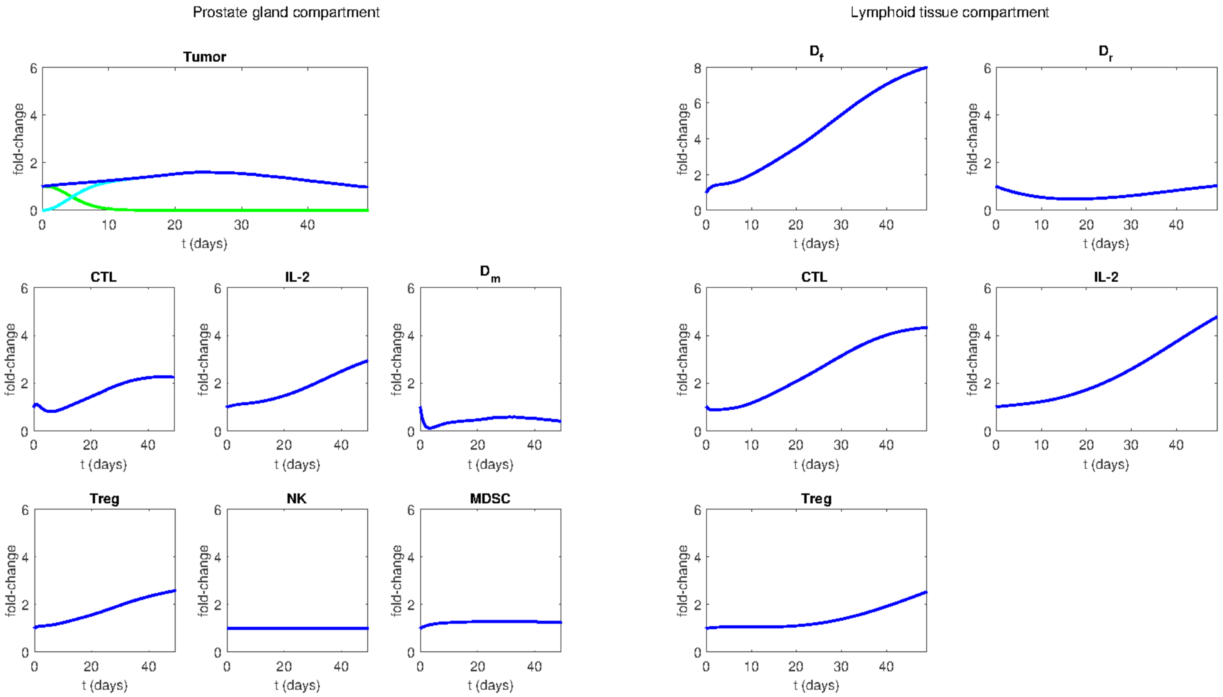

(a) (b)

Figure S6. **Model dynamics of androgen deprivation combined to ICB therapy.**

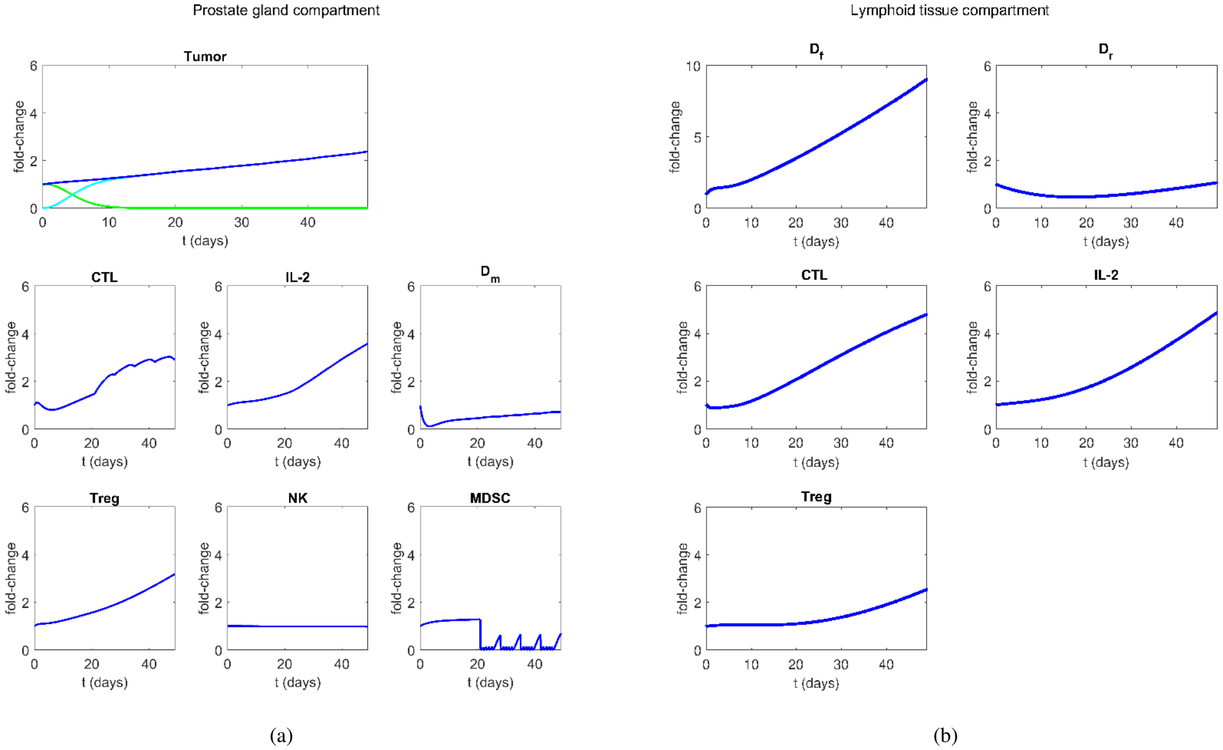

Figure S7. **Model dynamics of androgen deprivation combined to anti-MDSC therapy.**

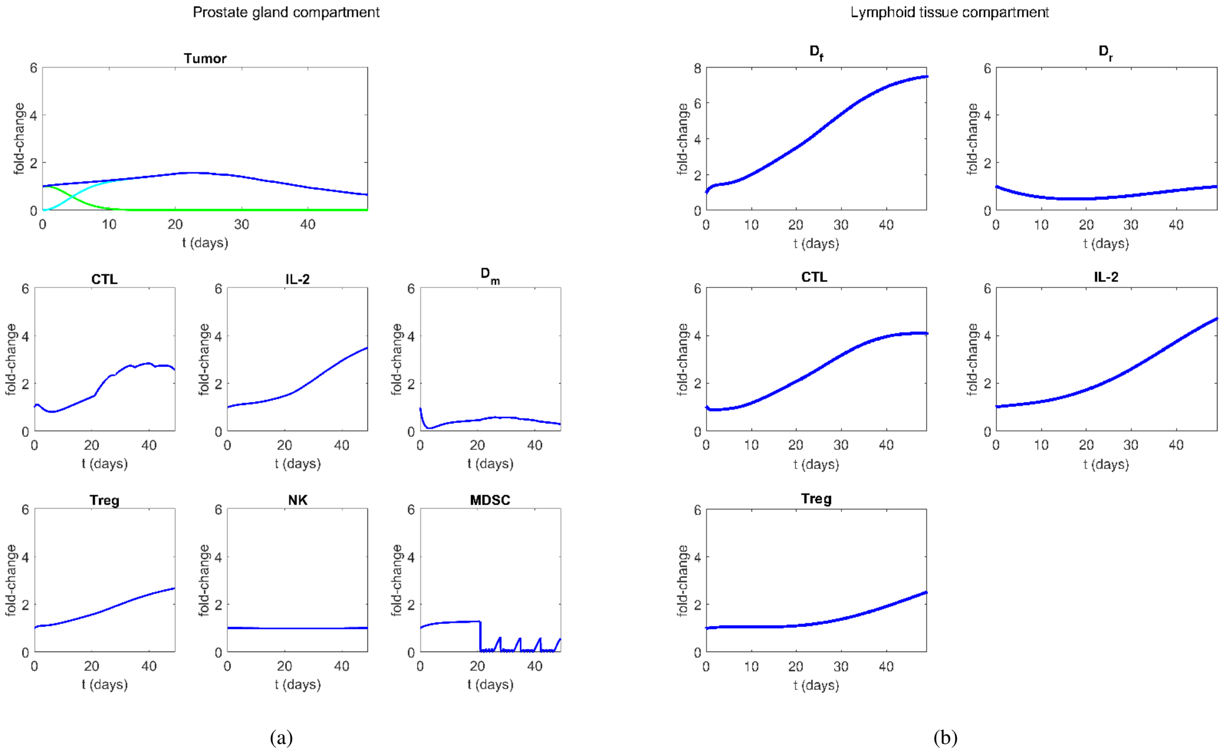

Figure S8. **Model dynamics of androgen deprivation combined to ICB and anti-MDSC" therapy.**

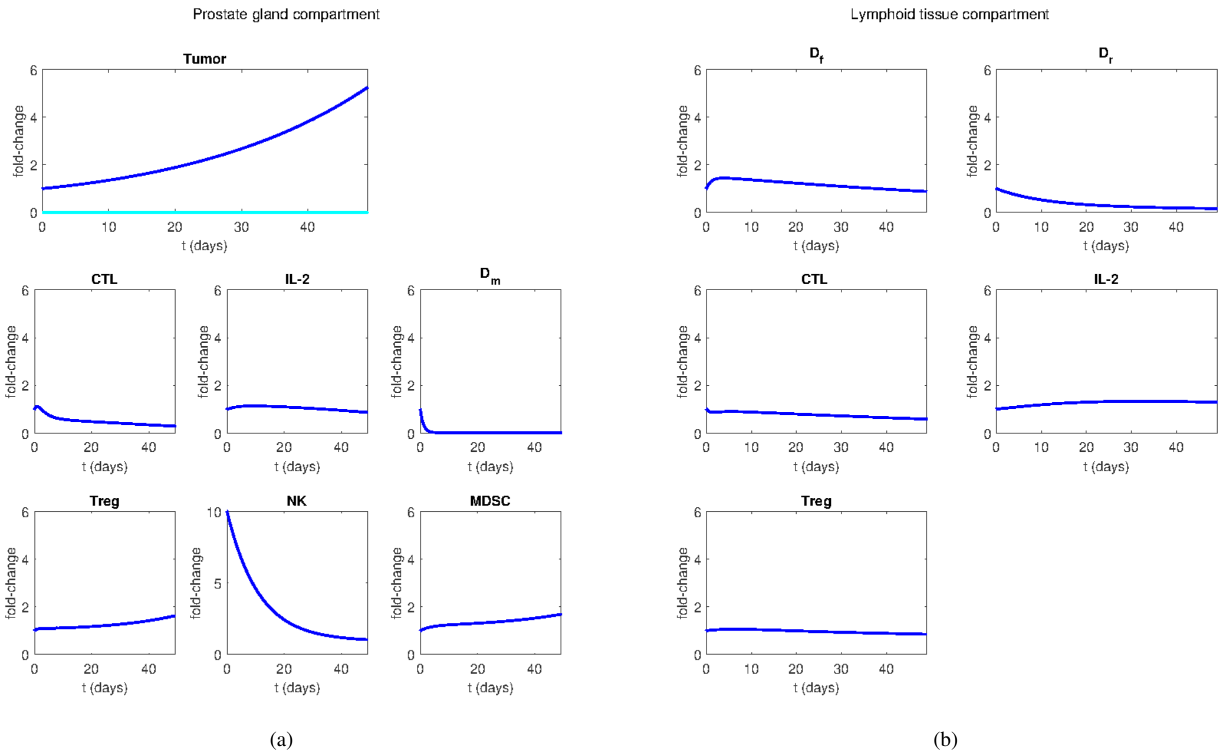

Figure S9. **Model dynamics of NK injection treatment.**

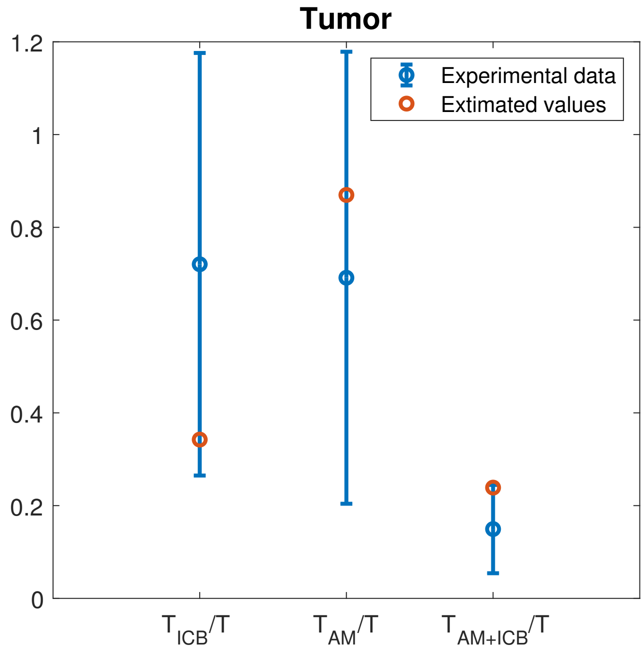

(a)

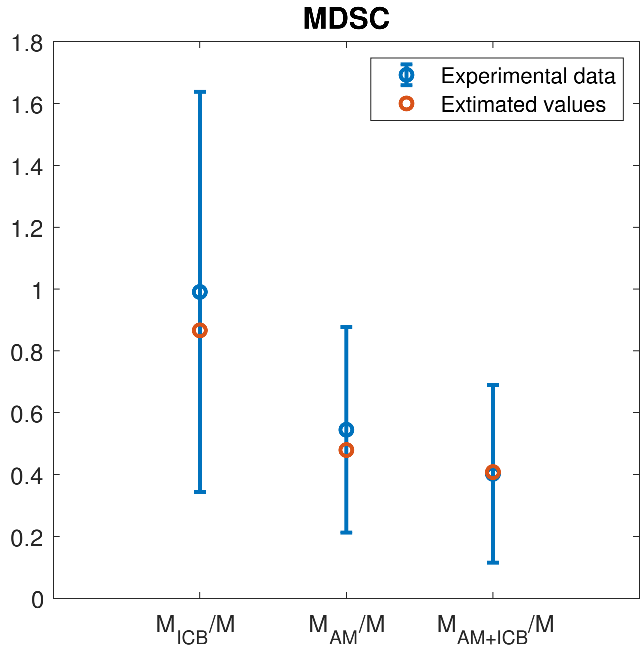

(b)

Figure S10. Data fitting for ICB and MDSC treatments. (a) Ratios of immuno-treated castration-resistant tumor (T) to untreated. (b) Ratios of MDSCs population (M) in immuno-treated prostate cancer to untreated. Described immunotherapy scenarios: ICB treatment (ICB), anti-MDSC treatment (AM) and ICB combined to anti-MDSC (AM+ICB). Experimental data[4] are plotted as blue points with the corresponding standard deviations (blue bar). Model simulation results are plotted as red points.

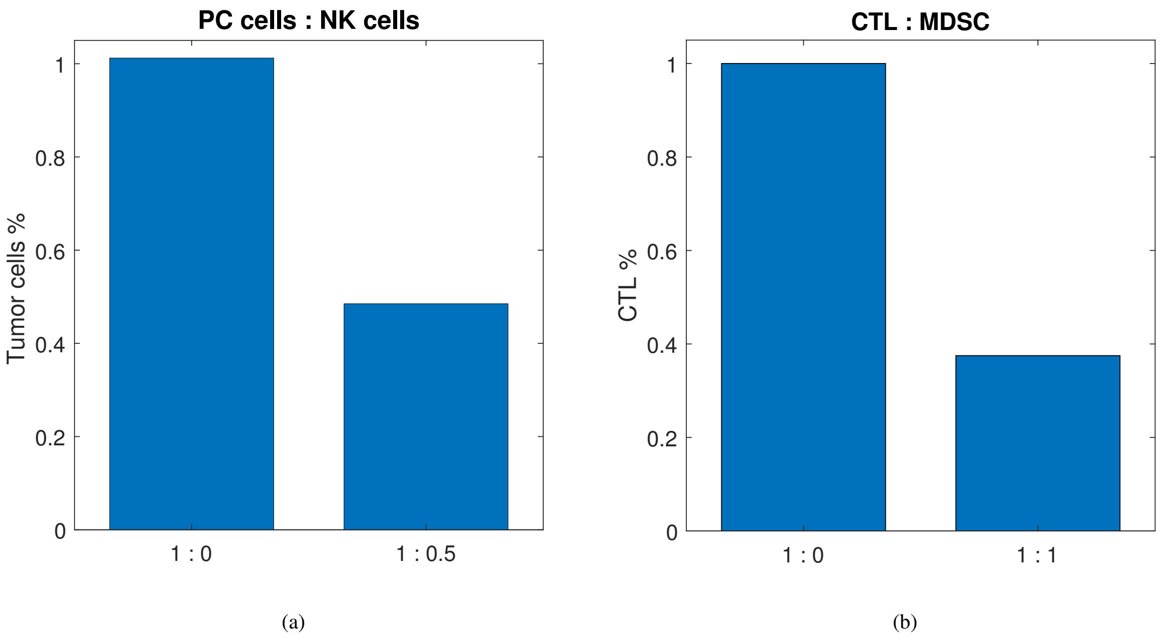

Figure S11. Model validation of data from *in vitro* experiments. Cell proportions reported on the x axis are specified at each graph top side. (a) 100% tumor cells is the control sample, reduced by 50% after NK cell co-culture[4]. (b) 100% CTLs is the control sample, reduced by 42% after MDSCs co-culture[3].

Treatments	Tumor inhibition percentage (after 4 weeks)
'V'	-0,51%
'V+AI'	-1,79%
'AI'	-2,18%
'V+NK'	-4,97%
'NK'	-7,04%
'V+AI+NK'	-8,32%
'V+AR'	-10,13%
'AR'	-10,45%
'AM'	-12,61%
'V+AI+AR'	-12,94%
'AI+NK'	-13,16%
'AI+AR'	-13,17%
'V+AM'	-13,47%
'AI+AM'	-14,56%
'V+AI+AM'	-14,66%
'AR+AM'	-21,77%
'V+AR+AM'	-22,24%
'AI+AR+AM'	-24,35%
'V+AI+AR+AM'	-24,77%
'ICB'	-26,06%
'AI+ICB'	-30,26%
'NK+ICB'	-31,98%
'CX'	-33,11%
'CX+V'	-33,60%
'CX+AI'	-34,06%
'CX+V+AI'	-34,24%
'CX+V+NK'	-36,31%
'CX+NK'	-37,12%
'CX+V+AI+NK'	-37,94%
'V+AR+NK'	-38,98%
'AI+NK+ICB'	-39,19%
'CX+AR'	-39,58%
'CX+V+AR'	-39,69%
'CX+AI+NK'	-39,99%
'CX+AI+AR'	-41,19%
'CX+V+AI+AR'	-41,29%
'CX+AM'	-41,77%
'CX+V+AM'	-42,40%
'CX+AI+AM'	-42,67%
'CX+V+AI+AM'	-43,05%
'AR+NK'	-43,43%
'ICB+AM'	-44,42%
'AR+ICB'	-44,93%
'V+AI+AR+NK'	-45,16%
'AI+AR+ICB'	-46,88%
'CX+AR+AM'	-47,58%
'CX+V+AR+AM'	-48,15%
'V+NK+AM'	-48,20%
'NK+AM'	-49,04%
'CX+AI+AR+AM'	-49,07%
'AI+AR+NK'	-49,18%
'AI+ICB+AM'	-49,31%
'CX+V+AI+AR+AM'	-49,58%
'V+AI+NK+AM'	-50,06%
'AI+NK+AM'	-52,56%
'V+ICB'	-54,27%
'V+NK+ICB'	-56,53%
'CX+V+AR+NK'	-58,21%
'V+AI+ICB'	-59,15%
'CX+AR+NK'	-60,83%
'CX+V+AI+AR+NK'	-62,09%
'V+AI+NK+ICB'	-62,17%
'CX+ICB'	-63,78%
'CX+AI+AR+NK'	-64,57%
'AR+ICB+AM'	-65,27%
'CX+V+NK+AM'	-65,36%
'CX+NK+AM'	-65,71%
'CX+NK+ICB'	-65,76%
'CX+V+AI+NK+AM'	-66,36%
'AI+AR+ICB+AM'	-66,64%
'CX+AI+ICB'	-66,64%
'V+AR+NK+AM'	-66,69%
'AR+NK+ICB'	-67,48%
'CX+AI+NK+AM'	-67,51%
'AR+NK+AM'	-68,90%
'CX+AI+NK+ICB'	-69,47%
'V+ICB+AM'	-69,49%
'NK+ICB+AM'	-69,60%
'V+AI+AR+NK+AM'	-70,28%
'AI+AR+NK+ICB'	-71,15%
'AI+AR+NK+AM'	-72,38%
'CX+ICB+AM'	-73,58%
'AI+NK+ICB+AM'	-73,95%
'V+AR+ICB'	-74,58%
'V+AI+ICB+AM'	-74,60%
'CX+AR+ICB'	-74,84%
'CX+V+ICB'	-75,40%
'V+AI+AR+ICB'	-75,75%
'CX+AI+AR+ICB'	-75,76%
'CX+V+NK+ICB'	-76,35%
'CX+AI+ICB+AM'	-76,75%
'CX+V+AR+NK+AM'	-77,43%
'CX+V+AI+ICB'	-78,47%
'CX+AR+NK+AM'	-78,74%
'CX+V+AI+NK+ICB'	-79,65%
'CX+V+AI+AR+NK+AM'	-79,71%
'CX+AI+AR+NK+AM'	-81,01%
'V+NK+ICB+AM'	-82,42%
'CX+AR+NK+ICB'	-83,04%
'V+AR+NK+ICB'	-83,49%
'CX+V+ICB+AM'	-83,58%
'CX+NK+ICB+AM'	-83,70%
'CX+AI+AR+NK+ICB'	-84,79%
'CX+AR+ICB+AM'	-85,13%
'V+AI+AR+NK+ICB'	-85,49%
'CX+AI+AR+ICB+AM'	-85,80%
'V+AI+NK+ICB+AM'	-85,82%
'CX+AI+NK+ICB+AM'	-86,16%
'CX+V+AI+ICB+AM'	-86,65%
'CX+V+AR+ICB'	-87,31%
'AR+NK+ICB+AM'	-87,74%
'CX+V+AI+AR+ICB'	-87,94%
'V+AR+ICB+AM'	-88,59%
'AI+AR+NK+ICB+AM'	-89,23%
'V+AI+AR+ICB+AM'	-89,26%
'CX+V+NK+ICB+AM'	-89,85%
'CX+V+AR+NK+ICB'	-91,01%
'CX+V+AI+NK+ICB+AM'	-91,91%
'CX+V+AI+AR+NK+ICB'	-92,04%
'CX+AR+NK+ICB+AM'	-93,40%
'CX+AI+AR+NK+ICB+AM'	-94,17%
'CX+V+AR+ICB+AM'	-94,46%
'CX+V+AI+AR+ICB+AM'	-94,82%
'V+AR+NK+ICB+AM'	-95,46%
'V+AI+AR+NK+ICB+AM'	-96,07%
'CX+V+AR+NK+ICB+AM'	-97,44%
'CX+V+AI+AR+NK+ICB+AM'	-97,78%

Figure S12. Therapy effect on tumor inhibition. The model predicted effect of every possible combination therapy. Therapies have been sorted by their tumor inhibition percentage compared to the untreated case, after 4 weeks of therapy. Treatments are named by the following abbreviations: Androgen Deprivation (CX), Anti-IL-2 (AI), Anti-Treg (AR), Anti-MDSC (AM), Vaccine (V), injection of NK cells (NK) and Immune-Checkpoint Blockade (ICB).

Parameter	Description	Value	Estimation Procedure
a_{CI}	Activation rate of IL-2 by CTLs	$5.84\ 10^{-2}\ day^{-1}$	taken from[2]
a_{DC}	Activation rate of CTLs by Dendritic cells	$4.24\ 10^{-1}\ day^{-1}$	optimized around the value from[2] +/-30%
a_{DfDr}	Transformation rate of functional Dendritic cells into regulatory Dendritic cells	$1.43\ 10^{-2}\ day^{-1}$	taken from[2]
a_{DR}	Activation rate of Treg by mature Dendritic cells	$1.06\ 10^{-1}\ day^{-1}$	taken from[2]
a_{DrR}	Activation rate of Tregs by regulatory Dendritic cells	$6.75\ 10^{-2}\ day^{-1}$	taken from[2]
a_{IC}	Maximal activation rate of CTLs by IL-2	$7.70\ 10^{2}\ day^{-1}$	unconstrained optimization
a_{IN}	Maximal activation rate of NK by IL-2	$7.43\ 10^{-2}\ day^{-1}$	optimized around the value from[8] after non-dimensionalization +/- 30%
a_{IR}	Activation rate of Treg by IL-2	$5.06\ 10^{-2}\ day^{-1}$	taken from[2]
a_{VD}	Activation rate of mature Dendritic cells by Vaccine	$8.08\ 10^{-1}\ day^{-1}$	optimized around the value from[2] +/-30%
a_{XD}	Activation rate of mature Dendritic cells by Tumor	$2.73\ 10^{-1}$	optimized around the value from[2] +/-30%
a_{XM}	Maximal activation rate of MDSC by Tumor	$6.70\ 10^{+2}\ day^{-1}$	optimized around the value from[6] after non-dimensionalization +/- 30%
a_{XR}	Activation rate of Treg by Tumor	$2.06\ 10^{-2}\ day^{-1}$	taken from[2]
k_{antiII}	Inhibition rate of IL-2 by anti-IL-2 drug	$7.14\ 10^{-1}\ day^{-1}$	taken from[2]
k_{antiMM}	Inhibition rate of MDSC by Cabozatinib drug	$7.57\ 10^{+1}\ day^{-1}$	optimized in $[0,100]$
k_{antiRR}	Inhibition rate of Treg by anti-Treg drug	$1.43\ day^{-1}$	taken from[2]
k_{CX}	Killing rate of tumor by CTLs	$6.55\ 10^{-4}\ day^{-1}$	optimized in $[0,k_{CX}^{Peng}]$, where k_{CX}^{Peng} is the k_{CX} estimated in[2]
k_{MC}	CTL cytotoxic effect MDSC-dependent inhibition	$9.62\ 10^{-2}\ day^{-1}$	optimized in $[0.001,10]$
k_{MN}	NK cytotoxic effect MDSC-dependent inhibition	3.88	optimized in $[0.001,10]$

Parameter	Description	Value	Estimation Procedure
k_{NX}	Maximal killing rate of Tumor by NK cells	$7.22\ 10^{-3}\ day^{-1}$	optimized in $[0.001, 10]/7$
k_{RC}	CTL cytotoxic effect Treg-dependent inhibition	$1.00\ 10^{-1}\ day^{-1}$	optimized in $[0, k_{RC}^{Peng}]$, where k_{RC}^{Peng} is the k_{RC} estimated in[2]
k_{RN}	NK cytotoxic effect Treg-dependent inhibition	2.07	optimized in $[0.001, 10]$
k_{XN}	Inactivation rate of NK by Tumor	$1.60\ 10^{-3}\ day^{-1}$	optimized in $[0.0001, 1]$
m_C	Migration rate of CTLs out of the lymphoid tissue	$4.29\ 10^{-1}\ day^{-1}$	optimized around the value from[2] +/-30%
m_D	Migration rate of mature Dendritic cells out of the prostate gland	$9.36\ 10^{-1}\ day^{-1}$	optimized around the value from[2] +/-30%
m_R	Migration rate of Treg out of the lymphoid tissue	$1.43\ 10^{-2}\ day^{-1}$	taken from[2]
p_C	Probability of migrating CTLs to reach the prostate gland	0.5	taken from[2]
p_D	Probability of migrating mature dendritic cells to reach the lymphoid tissue	0.5	taken from[2]
p_R	Probability of migrating Treg cells to reach the prostate gland	0.5	taken from[2]
r_M	Mutation rate of ADPC into AIPC	$8.11\ 10^{-1}\ day^{-1}$	optimized around the value from[2] +/-30%
r_{p1}	Proliferation rate of Androgen Dependent Prostate Cancer cells (ADPC)	$3.63\ 10^{-2}\ day^{-1}$	optimized around the value from[2] +/-30%
r_{p2}	Proliferation rate of Androgen Independent Prostate Cancer cells (AIPC)	$1.70\ 10^{-1}\ day^{-1}$	optimized around the value from[2] +/-30%
s_I	IL-2 saturation level for CTL clonal expansion	$7.72\ 10^{4}$	optimized around the value from[7] +/-30%
s_M	Tumor cells saturation level for MDSC clonal expansion	$1.82\ 10^{+4}$	optimized around the value from[6]after non-dimensionalization +/- 30%
s_N	IL-2 saturation level for NK clonal expansion	$1.49\ 10^{+2}$	optimized around the value from[8] after non-dimensionalization +/- 30%
δ_{ICB}	Effect of ICB drugs on CTL killing rate	$1.09\ 10^{+1}$	optimized in $[1, 100]$

Parameter	Description	Value	Estimation Procedure
λ_A	Decay rate for Androgens under androgen deprivation therapy	$9.90\ 10^{-2}\ day^{-1}$	computed as explained in the description of Eq. (9)
λ_{AI}	Decay rate for anti-IL-2	$9.90\ 10^{-2}\ day^{-1}$	computed as explained in the description of Eq. (9) by considering the correspondent anti-IL-2 drug half-life
λ_{AM}	Decay rate for Cabozatinib drug	$4.75\ day^{-1}$	computed as explained in the description of Eq. (9) by considering the correspondent anti-MDSC drug half-life
λ_{AR}	Decay rate for anti-Treg	$9.90\ 10^{-2}\ day^{-1}$	computed as explained in the description of Eq. (9) by considering the correspondent anti-Treg drug half-life
λ_{ICB}	Decay rate for ICB	$1.39\ 10^{-1}\ day^{-1}$	computed as explained in the description of Eq. (9) by considering the correspondent ICB drug half-life
λ_V	Decay rate for Vaccine	$9.90\ 10^{-2}\ day^{-1}$	computed as explained in the description of Eq. (9) by considering the correspondent vaccine half-life
μ_1	ADPC death rate	$1.01\ 10^{-2}\ day^{-1}$	optimized around the value from[2] +/-30%
μ_2	AIPC death rate	$1.48\ 10^{-1}\ day^{-1}$	optimized around the value from[2] +/-30%
μ_C	CTL death rate	$1.46\ 10^{-1}\ day^{-1}$	optimized around the value from[2] +/-30%
μ_D	Dendritic cell death rate	$9.29\ 10^{-2}\ day^{-1}$	taken from[2]
μ_I	IL-2 death rate	$3.05\ 10^{-2}\ day^{-1}$	taken from[2]
μ_M	MDSC death rate	$2.94\ 10^{-1}\ day^{-1}$	optimized around the value from[6]after non-dimensionalization +/- 30%
μ_R	Treg death rate	$8.07\ 10^{-2}\ day^{-1}$	taken from[2]
μ_N	NK cell death rate	$9.00\ 10^{-2}\ day^{-1}$	calculated as in[8] (see NK equation (5) for details)
ρ_M	MDSC source	$3.24\ 10^{-1}$	optimized around the value from[6]after non-dimensionalization +/- 30%
ρ_N	NK cell source	$9.00\ 10^{-2}\ day^{-1}$	computed by imposing the steady state as explained in NK Eq. (5)

Table S1. Table of model parameters.

References

1. A Domogala, J. A. Madrigal, A. Saudemont. *Natural Killer Cell Immunotherapy: From Bench to Bedside*. Frontiers in Immunology, 6:264, 2015.
2. H. Peng, W. Zhao, H. Tan, Z. Ji, J. Li, K. Li, X. Zhou. *Prediction of treatment efficacy for prostate cancer using a mathematical model*. Scientific Reports, 6:21599, 2016.
3. A. J. Garcia, M. Ruscetti, T. L. Arenzana, L. M. Tran, D. Bianci-Frias, D. E. Sybert, S. J. Priceman, L. Wu, P. S. Nelson, S. T. Smale, H. Wu. *Pten Null Prostate Epithelium Promotes Localized Myeloid-Derived Suppressor Cell Expansion and Immune Suppression during Tumor Initiation and Progression*. Molecular and Cellular Biology, 34(11):2017-2028, 2014.
4. X. Lu, J. W. Horner, E. Paul, x. Shang, P. Troncoso, P. Deng, S. Jiang, q. Chang, D. J Spring, P. Sharma, J. A. Zebala, D. Y. Maeda, Y. A. Wang, R. A. DePinho. *Effective combinatorial immunotherapy for castration-resistant prostate cancer*. Nature, 543:728, 2017.
5. S. J. Lin, F. J. Chou, L. Li, C. Y. Lin, S. Yeh, C. Chang. *Natural killer cells suppress enzalutamide resistance and cell invasion in the castration resistant prostate cancer via targeting the androgen receptor splicing variant 7 (ARv7)*. Cancer Letters, 398:62-69, 2017.
6. S. P. Shariatpanahi, S. P. Shariatpanahi, K. Madjidzadeh, M. Hassan, M. Abedi-Valugerdi. *Mathematical modeling of tumor-induced immunosuppression by myeloid-derived suppressor cells: Implications for therapeutic targeting strategies*. Journal of Theoretical Biology, 442:1-10, 2018.
7. E. M. Rutter, Y. Kuang. *Global dynamics of a model of joint hormone treatment with dendritic cell vaccine for prostate cancer*. Discrete and Continuous Dynamical Systems - Series B, 22(3):1001-1021, 2017.
8. L. de Pillis, T. Caldwell, E. Sarapata, H. Williams. *Mathematical modeling of regulatory T cell effects on renal cell carcinoma treatment*. Discrete and Continuous Dynamical Systems - Series B, 18:915-943, 2013.
