# Supplementary figures and images for: A QSP model of prostate cancer immunotherapy to identify effective combination therapies
Source: Sci Rep. 2020 Jun 3;10:9063. doi: 10.1038/s41598-020-65590-0 (PMC7270132; doi:10.1038/s41598-020-65590-0)

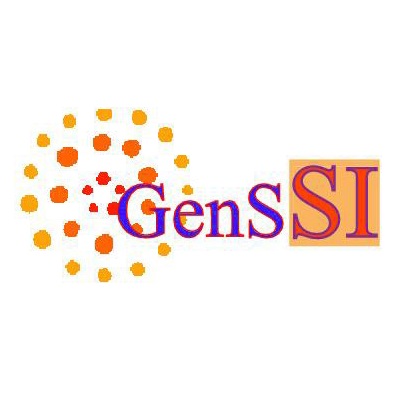

Supplement: Supplementary file 2 [file 41598_2020_65590_MOESM2_ESM.zip › ColettiEtAl_SupplementaryFile2/Docu/input/GenSSI-logo-square.jpg]

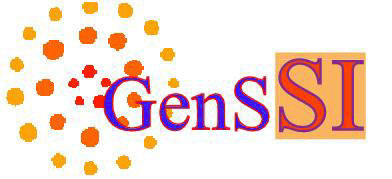

Supplement: Supplementary file 2 [file 41598_2020_65590_MOESM2_ESM.zip › ColettiEtAl_SupplementaryFile2/Docu/input/GenSSI-logo.jpg]
