# Supplementary material for: A QSP model of prostate cancer immunotherapy to identify effective combination therapies
Source: Sci Rep. 2020 Jun 3;10:9063. doi: 10.1038/s41598-020-65590-0 (PMC7270132; doi:10.1038/s41598-020-65590-0)
Supplement: Supplementary file 6 [file 41598_2020_65590_MOESM6_ESM.pdf]

Supplementary information

***Supplementary File 1*:** supplementary figures and tables.
***Supplementary File 2*:** Matlab codes for the structural identifiability analysis.
***Supplementary File 3*:** results of the LSA performed on tumor size for each experimental scenario.
***Supplementary File 4*:** results of the GSA performed on tumor size for each experimental scenario.
***Supplementary File 5*:** table of Bliss Combination Indices (BCIs).
***Supplementary File 6*:** list of supplementary files.
